# Recombination fraction in pre-recombinant inbred lines (PRERIL) - revisiting a century old problem in genetics

**DOI:** 10.1186/s12864-024-10699-z

**Published:** 2024-09-02

**Authors:** Shizhong Xu, José Osorio y Fortéa

**Affiliations:** 1 Department of Botany and Plant Sciences, University of California, Riverside, CA 92521 USA; 2 Limagrain Vegetable Seeds, Vilmorin & Cie, 28 Route d’Ennezat, Chappes, Zip 63720 France

**Keywords:** Computer generated transition matrix, Markov chains, Recombinant inbred lines, Recurrent equations

## Abstract

**Background:**

Traditional recombinant inbred lines (RILs) are generated from repeated self-fertilization or brother-sister mating from the F_1_ hybrid of two inbred parents. Compared with the F_2_ population, RILs cumulate more crossovers between loci and thus increase the number of recombinants, resulting in an increased resolution of genetic mapping. Since they are inbred to the isogenic stage, another consequence of the heterozygosity reduction is the increased genetic variance and thus the increased power of QTL detection. Self-fertilization is the primary form of developing RILs in plants. Brother-sister mating is another way to develop RILs but in small laboratory animals. To ensure that the RILs have at least 98% of homozygosity, we need about seven generations of self-fertilization or 20 generations of brother-sister mating. Prior to homozygosity, these lines are called pre-recombinant inbred lines (PRERIL). Phenotypic values of traits in PRERILs are often collected but not used in QTL mapping. To perform QTL mapping in PRERILs, we need the recombination fraction between two markers at generation *t* for *t* < 7 (selfing) or *t* < 20 (brother-sister mating) so that the genotypes of QTL flanked by the markers can be inferred.

**Results:**

In this study, we developed formulas to calculate the recombination fractions of PRERILs at generation *t* in self-fertilization, brother-sister mating, and random mating. In contrast to existing works in this topic, we used computer code to construct the transition matrix to form the Markov chain of genotype array between consecutive generations, the so-called recurrent equations.

**Conclusions:**

We provide R functions to calculate the recombination fraction using the newly developed recurrent equations of ordered genotype array. With the recurrent equations and the R code, users can perform QTL mapping in PRERILs. Substantial time and effort can be saved compared with QTL mapping in RILs.

**Supplementary Information:**

The online version contains supplementary material available at 10.1186/s12864-024-10699-z.

## Background

Herbert Spencer Jennings (1868–1947), an American Professor, was the first geneticist to study the behaviors of recombinant inbred lines (RILs) [9]. At that time, recombinant inbred lines had not been conceptualized. Jennings [8, 9] called the two copies of a single Mendelian locus a pair of Mendelian characters while called alleles from two loci two pairs of Mendelian characters, where Mendelian factors are referred to as genetic units passed from parents to offspring. Jennings described the process of generating RILs as repeated self-fertilization, starting from the F_1_ hybrid. Although his work was general so that the RILs can start with any genotypes, his purpose was to investigate the proportions of genotypes of two linked loci at generation *t + *1 given the proportions of the genotypes at generation *t*. This works was an extension of his previous work for a single locus [8]. The problems investigated by Jennings are very challenging [17]. In addition to self-fertilization, Jennings [8, 9] investigated many other mating schemes, including random mating, brother-sister mating, parent–offspring mating, and even selection and assortative mating. At the same time, it was hard to follow. The article was almost all in theory with little context. We may want to know more about the interest at the time of such schemes as parent-by-offspring mating in which each individual is used for two, and exactly two, successive generations [17].

Jennings’ [8, 9] work was fundamental but very disorganized in its written form, unfortunately. It is not until Robbins [16] who redescribed Jennings’ work in a clear and well organized manner, that Jennings’ [9] work became well-known. Two mating systems (random mating and self-fertilization) introduced by Jennings and reintroduced by Robbins will be discussed in this study. However, we mainly cited the work by Robbins [14–16]. Both Jennings and Robbins defined the parameter of interest as linkage ratio denoted by *r*. But their *r* and the *r* in modern genetics are entirely different. The *r* defined as linkage ratio is a relative number of the parental types of gametes compared with the recombinant types of gametes [9]. The *r* defined as the recombination fraction in modern genetics is a proportion of the recombinant gametes over all possible gametes in a population of interest. To avoid any potential confusion, we denote the recombination fraction in modern genetics by $\theta$  to avoid using *r* as the recombination fraction.

Robbins’ random mating recurrent equations are clearer. His equations lead to the proportions of the four types of gametes expressed as functions of *r* and the number of generations of random mating. He concluded that (1) in random mating, the effect of incomplete linkage between two factors is only temporary and (2) continued random mating results in a population in which the distribution of “B” factors among the “A” and “a” factors is the same as the distribution of the “b” factors among the “A” and “a” factors. The second conclusion is simply the statistical independence between the two factors or linkage equilibrium after many generations of random mating. In fact, the two conclusions imply the same consequence in modern quantitative genetics: genetic correlation caused by incomplete linkage is temporary [12].

Robbins [16] reinvestigated all problems raised by Jennings [9] and extended the work into a higher level. Especially for the random mating system where he, after extensive derivation, developed a functional relationship of the gametic frequencies to the initial gametic frequencies using the sum of geometric series. As demonstrated in Supplementary Note S3, the functional relationship of the recombination fraction of Robbins is identical to the functional relationship developed by Darvasi and Soller [4] who used an extremely simple method. Darvasi and Soller [4] called the lines generated from such a repeated random mating scheme the advanced intercross lines (AIL). They first derived the recurrent equation for the recombination fraction starting with the F_1_ hybrid of a cross between two inbred lines. From the recurrent equation, they expressed the recombination fraction at generation *t* as a function of the recombination fraction in the original population,

1 $$\theta_t=\frac12\left[1-\left(1-2\theta\right)\left(1-\theta\right)^{t-2}\right]$$

where $t\geq2$ with $t=2$ being the F_2 _population.

Robbins’ [16] other contribution to the subject was to reinvestigate the recurrent genotypic frequencies in the self-fertilization system. He pooled the $4\times4=16$ total genotypes with phase information into 10 distinguished unphased genotypes. The recurrent equations were much cleaner than those given by Jennings [9], although Robbins inherited Jennings’s notation system, e.g., using the same *r* to represent the linkage ratio and the same *p*, *q*, *s*, *t* notation to denote the four gametic frequencies in the random mating system. Robbins did not provide the asymptotic recombination fraction after infinite number of generations with self-fertilization.

Haldane and Waddington [6] developed the recombination fractions at the equilibrium stage after infinite number of self-fertilization and brother-sister mating. Haldane and Waddington [6] combined some of the 10 unphased genotypes proposed by Robins [16] into a common class and yielded 5 composite genotypes. They delt with only 5 recurrent equations, significantly reduced the computational complexity. The major contribution of the Haldane and Waddington’s study [6] was the brother-sister matting system for linkage analysis, which was not touched by previous authors for two pairs of linked characters. Haldane and Waddington [6] developed the $10\times10=100$ recurrent equations for the genotypes of the sibling pairs. The absorption of the original $16\times16=256$ fully phased recurrent equations into the $10\times10=100$ unphased recurrent equations represents a substantial reduction in computation. The recurrent equations convert the frequencies of the 100 genotype combinations from the previous generation to the genotype frequencies of the current generation using a $10\times10$ transition matrix in the Markov chain system.

Other than the recurrent equation of the recombination fraction developed by Darvasi and Soller [4], none of the previous works reported the recombination fraction before the consecutive mating systems reach equilibrium. The recurrent equations for genotype frequencies under self-fertilization and brother-sister mating were all derived manually, which are prone to error when a computer program code is written. Broman [2] extended the asymptotic recombination fraction of RILs of brother-sister mating from an 8-way crosses and showed that the final recombination fraction is

2 $$\rho_{SW}=\frac{7\theta}{1+6\theta}$$

No recurrent equations are provided to determine the recombination fraction before the lines reach the equilibrium value. The purposes of this study are to present (1) a derivation of the recombination fraction at generation ($t<\infty$) before the system reaches the equilibrium, (2) a computer code generated transition matrix for recurrent equations of genotype frequencies. Relevant background knowledge and recombination fraction at generation ($t<\infty$) from works of previous authors are given in the Supplementary Note S1, Note S2 and Note S3.

## Methods

### Basic definition

Consider two linked loci (A and B) with a recombination fraction of $\theta$  for $0<\theta<0.5$. Define the diploid genotypes of the two inbred parents that initiate the F_1_ hybrid by *AB/AB* and *ab/ab*, respectively. The genotype of the F_1_ hybrid is *AB/ab*. In each genotype, the maternal and paternal gametes are separated by a slash. The four possible gametes from this F_1_ hybrid are *AB, Ab, aB* and *ab* with probabilities $\frac12\left(1-\theta\right)$, $\frac12\theta$, $\frac12\theta$ and $\frac12\left(1-\theta\right)$, respectively. The gametes of the F_1_ hybrid make the genotypes of the F_2_ population. Therefore, the recombination fraction of the F_2_ generation is

3 $$\theta_2=\frac{\Pr\left(Ab\right)+\Pr\left(aB\right)}{\Pr\left(AB\right)+\Pr\left(aB\right)+\Pr\left(Ab\right)+\Pr\left(ab\right)}=\frac{{\displaystyle\frac12}\theta+{\displaystyle\frac12}\theta}{{\displaystyle\frac12}\left(1-\theta\right)+{\displaystyle\frac12}\theta+{\displaystyle\frac12}\theta+{\displaystyle\frac12}\left(1-\theta\right)}=\theta$$

The mating system will start with *t*=1, i.e., the F_1_ generation, from which the recurrent equations of genotypes will be developed. The 4×4=16 possible genotypes in the F_2_ population are shown in Table 1 below.

**Table 1:** The 16 possible genotypes of the F_2_  population from the F_1_ hybrid with genotype *AB/ab*

Female\Male	*AB* $\tfrac{1}{2}(1 - \theta )$	*Ab* $\tfrac{1}{2}\theta$	*aB* $\tfrac{1}{2}\theta$	*ab* $\tfrac{1}{2}(1 - \theta )$
*AB* $\tfrac{1}{2}(1 - \theta )$ *)*	*AB/AB * $\tfrac{1}{4}{(1 - \theta )^2}$	*AB/Ab * $\tfrac{1}{4}\theta (1 - \theta )$	*AB/aB * $\tfrac{1}{4}\theta (1 - \theta )$	*AB/ab * $\tfrac{1}{4}{(1 - \theta )^2}$
*Ab* $\tfrac{1}{2}\theta$	*Ab/AB * $\tfrac{1}{4}\theta (1 - \theta )$	*Ab/Ab * $\tfrac{1}{4}{\theta^2}$	*Ab/aB * $\tfrac{1}{4}{\theta^2}$	*Ab/ab * $\tfrac{1}{4}\theta (1 - \theta )$
*aB* $\tfrac{1}{2}\theta$	*aB/AB * $\tfrac{1}{4}\theta (1 - \theta )$	*aB/Ab * $\tfrac{1}{4}{\theta^2}$	*aB/aB * $\tfrac{1}{4}{\theta^2}$	*aB/ab * $\tfrac{1}{4}\theta (1 - \theta )$
*ab* $\tfrac{1}{2}(1 - \theta )$	*ab/AB * $\tfrac{1}{4}{(1 - \theta )^2}$	*ab/Ab * $\tfrac{1}{4}\theta (1 - \theta )$	*ab/aB * $\tfrac{1}{4}\theta (1 - \theta )$	*ab/ab * $\tfrac{1}{4}{(1 - \theta )^2}$

In the current literature, the recombination fraction is often denoted by *r*. However, Jennings [9] and Robbins (1917) defined *r* as a linkage ratio parameter with an entirely different interpretation. They set each of the recombinant gametes to 1, and each of the parental gametes to *r* relative to the recombinant gamete. The relative contributions of the four gametes are $r$ from $AB$ or $ab$, and $1$ from $Ab$ or $aB$. To avoid notational confusion, we denote the recombination fraction by $\theta$. The relationship between $\theta$ and $r$ is

4 $$\theta = \frac{1}{1 + r}{\text{ or }}r = \frac{1 - \theta }{\theta }$$

Starting from Table 1, the recurrent equations of genotype and gamete frequencies are developed for self-fertilization, brother-sister mating, and random mating. These recurrent equations allow us to calculate the recombination fractions of PRERILs at generation *t*. We start with self-fertilization, then brother-sister mating, and finally random mating. We assume that the recombination fractions are the same for the male and female gametes. Haldane and Waddington [6] denoted the recombination fraction for the female gamete by $\beta$ and for the male gamete by $\delta$. They intended to cover insects, which often have different recombination fractions between sexes. We do not differentiate the male and female gametes and thus the results of this study only apply to diploid plants and diploid animals where $\beta = \delta = \theta$ is the recombination fraction.

The 16 fully phased genotypes in Table 1 are re-arranged into a column vector with the order shown in Table 2. Verbally, the four genotypes of the first row in Table 1 become the first four genotypes in the $16 \times 1$ vector of Table 2. Gametic probabilities that each of the 16 fully phased genotypes can produce are presented in Table 2 also. For example, entry 2 of Table 2 shows the genotype of *AB/Ab* and the probabilities of producing the four possible gametes from this genotype are 0.5 for *AB*, 0.5 for *Ab*, 0 for *aB* and 0 for *ab*. Another example is entry 7, which is genotype *Ab/aB*. This genotype can produce all four gametes with the following probabilities: $\tfrac{1}{2}\theta$ for *AB*, $\tfrac{1}{2}(1 - \theta )$ for *Ab*, $\tfrac{1}{2}(1 - \theta )$ for *aB* and $\tfrac{1}{2}\theta$ for *ab*. The $16 \times 4$ gametic probability table (the *H* matrix) is the key to form the recurrent equations for genotypes across generations in all mating systems discussed in this study. This *H* matrix can be generated automatically via a computer program.

**Table 2:** Gametic probability table (the *H* matrix) from each of the 16 fully phased genotypes

Entry	Genotype	*AB*	*Ab*	*aB*	*ab*
1	*AB/AB*	1	0	0	0
2	*AB/Ab*	1/2	1/2	0	0
3	*AB/aB*	1/2	0	1/2	0
4	*AB/ab*	$\tfrac{1}{2}(1 - \theta )$	$\tfrac{1}{2}\theta$	$\tfrac{1}{2}\theta$	$\tfrac{1}{2}(1-\theta)$
5	*Ab/AB*	1/2	1/2	0	0
6	*Ab/Ab*	0	1	0	0
7	*Ab/aB*	$\tfrac{1}{2}\theta$	$\tfrac{1}{2}(1 - \theta )$	$\tfrac{1}{2}(1 - \theta )$	$\tfrac{1}{2}\theta$
8	*Ab/ab*	0	1/2	0	1/2
9	*aB/AB*	1/2	0	1/2	0
10	*aB/Ab*	$\tfrac{1}{2}\theta$	$\tfrac{1}{2}(1 - \theta )$	$\tfrac{1}{2}(1 - \theta )$	$\tfrac{1}{2}\theta$
11	*aB/aB*	0	0	1	0
12	*aB/ab*	0	0	1/2	1/2
13	*ab/AB*	$\tfrac{1}{2}(1 - \theta )$	$\tfrac{1}{2}\theta$	$\tfrac{1}{2}\theta$	$\tfrac{1}{2}(1 - \theta )$
14	*ab/Ab*	0	1/2	0	1/2
15	*ab/aB*	0	0	1/2	1/2
16	*ab/ab*	0	0	0	1

### Recurrent equations of genotype frequencies for self-fertilization

Starting from the F_2_ population with recombination fraction $\theta$, after more than eight generations of continuous self-fertilization, the recombination fraction will reach its equilibrium value [6],

The recombination fraction at generation $t < 8$ can be obtained via recurrent equations of genotypes. We will derive the recurrent equations using matrix algebra. Matrix *H* is all what we need to build the $16 \times 16$ transition matrix *P*, from which the recurrent equations for computing the frequencies of the 16 genotypes are formed. We denote the genotype frequencies at generation *t* by a $16 \times 1$ vector ${G_t}$. The genotypic frequencies at generation $t + 1$ are computed from the frequencies at generation *t*,

5 $${G_{t + 1}} = P{G_t}$$

for $t \geqslant 1$, where $P$ is the transition matrix. The sequences of *G* across generations forms a Markov chain with transition matrix *P*. The above recurrent equations can be further manipulated into

6 $${G_{t + 1}} = P{G_t} = {P^2}{G_{t - 1}} = {P^3}{G_{t - 2}} = \cdots = {P^t}{G_1}$$

The genotype frequencies are functions of the genotype frequencies of the initial population (the ${{\text{F}}_1}$ individual) with genotype $AB/ab$ and $ab/AB$, which are the 4th and the 13th genotypes (see Table 2). Therefore, the initial genotype frequency vector has all elements being zero except ${G_1}[4] = {G_1}[13]$ $= 1/2$.

We now build the $16 \times 16$ transition matrix *P* one column at a time via matrix algebra and through computer programming. Let ${P_{k}}$ be the *k*th column of matrix ${P}$ for $k = 1, \cdots ,16$. Let ${h_k}$ be the *k*th row of matrix *H* for $k = 1, \cdots ,16$ (Table 2). The *k*th column of matrix *P* is

7 $$P_{\cdot k}=\text{vec}(h_k^Th_k)$$

where ${\text{vec}}(X)$ is a vectorization operator for matrix *X*. For example, ifthenwhich is a column vector. Let us use the following three genotypes as examples to demonstrate the three columns of matrix *P*. For the first genotype (entry 1 of Table 2), we generate the following matrix,

$$h_1^T{h_1} = \left[ {\begin{array}{*{20}{c}} 1 \\ 0 \\ 0 \\ 0 \end{array}} \right]\left[ {\begin{array}{*{20}{c}} 1&0&0&0 \end{array}} \right] = \left[ {\begin{array}{*{20}{c}} 1&0&0&0 \\ 0&0&0&0 \\ 0&0&0&0 \\ 0&0&0&0 \end{array}} \right]$$

Similarly, we can generate the third genotype (entry 3 of Table 2) as

$$h_3^T{h_3} = \left[ {\begin{array}{*{20}{c}} {\tfrac{1}{2}} \\ 0 \\ {\tfrac{1}{2}} \\ 0 \end{array}} \right]\left[ {\begin{array}{*{20}{c}} {\tfrac{1}{2}}&0&{\tfrac{1}{2}}&0 \end{array}} \right] = \left[ {\begin{array}{*{20}{c}} {\tfrac{1}{4}}&0&{\tfrac{1}{4}}&0 \\ 0&0&0&0 \\ {\tfrac{1}{4}}&0&{\tfrac{1}{4}}&0 \\ 0&0&0&0 \end{array}} \right]$$

and the seventh genotype (entry 7 of Table 2) as

$$h_7^T{h_7} = \left[ {\begin{array}{*{20}{c}} {\tfrac{1}{2}\theta } \\ {\tfrac{1}{2}(1 - \theta )} \\ {\tfrac{1}{2}(1 - \theta )} \\ {\tfrac{1}{2}\theta } \end{array}} \right]\left[ {\begin{array}{*{20}{c}} {\tfrac{1}{2}\theta }&{\tfrac{1}{2}(1 - \theta )}&{\tfrac{1}{2}(1 - \theta )}&{\tfrac{1}{2}\theta } \end{array}} \right] = \left[ {\begin{array}{*{20}{c}} {\tfrac{1}{4}{\theta^2}}&{\tfrac{1}{4}\theta (1 - \theta )}&{\tfrac{1}{4}\theta (1 - \theta )}&{\tfrac{1}{4}{\theta^2}} \\ {\tfrac{1}{4}\theta (1 - \theta )}&{\tfrac{1}{4}{{(1 - \theta )}^2}}&{\tfrac{1}{4}{{(1 - \theta )}^2}}&{\tfrac{1}{4}\theta (1 - \theta )} \\ {\tfrac{1}{4}\theta (1 - \theta )}&{\tfrac{1}{4}{{(1 - \theta )}^2}}&{\tfrac{1}{4}{{(1 - \theta )}^2}}&{\tfrac{1}{4}\theta (1 - \theta )} \\ {\tfrac{1}{4}{\theta^2}}&{\tfrac{1}{4}\theta (1 - \theta )}&{\tfrac{1}{4}\theta (1 - \theta )}&{\tfrac{1}{4}{\theta^2}} \end{array}} \right]$$

All the $h_k^T{h_k}$ matrices for $k = 1, \cdots ,16$ will be generated this way. From the $h_k^T{h_k}$ matrix, we build the *k*th column of the $16 \times 16$ transition matrix $P$. For the three genotypes demonstrated above, we obtain the following three column vectors,

$${P_{\cdot1}} = {\text{vec}}(h_1^T{h_1}) = \left[ {\begin{array}{*{20}{c}} 1 \\ 0 \\ 0 \\ 0 \\ 0 \\ 0 \\ 0 \\ 0 \\ 0 \\ 0 \\ 0 \\ 0 \\ 0 \\ 0 \\ 0 \\ 0 \end{array}} \right], \, {P_{\cdot3}} = {\text{vec}}(h_3^T{h_3}) = \left[ {\begin{array}{*{20}{c}} {\tfrac{1}{4}} \\ 0 \\ {\tfrac{1}{4}} \\ 0 \\ 0 \\ 0 \\ 0 \\ 0 \\ {\tfrac{1}{4}} \\ 0 \\ {\tfrac{1}{4}} \\ 0 \\ 0 \\ 0 \\ 0 \\ 0 \end{array}} \right], \, {P_{\cdot7}} = {\text{vec}}(h_7^T{h_7}) = \left[ {\begin{array}{*{20}{c}} {\tfrac{1}{4}{\theta^2}} \\ {\tfrac{1}{4}\theta (1 - \theta )} \\ {\tfrac{1}{4}\theta (1 - \theta )} \\ {\tfrac{1}{4}{\theta^2}} \\ {\tfrac{1}{4}\theta (1 - \theta )} \\ {\tfrac{1}{4}{{(1 - \theta )}^2}} \\ {\tfrac{1}{4}{{(1 - \theta )}^2}} \\ {\tfrac{1}{4}\theta (1 - \theta )} \\ {\tfrac{1}{4}\theta (1 - \theta )} \\ {\tfrac{1}{4}{{(1 - \theta )}^2}} \\ {\tfrac{1}{4}{{(1 - \theta )}^2}} \\ {\tfrac{1}{4}\theta (1 - \theta )} \\ {\tfrac{1}{4}{\theta^2}} \\ {\tfrac{1}{4}\theta (1 - \theta )} \\ {\tfrac{1}{4}\theta (1 - \theta )} \\ {\tfrac{1}{4}{\theta^2}} \end{array}} \right]$$

The 16 column vectors form the transition matrix *P*,

$$P = \left[ {\begin{array}{*{20}{c}} {{P_{1}}}&{{P_{2}}}&{{P_{3}}}& \cdots &{{P_{16}}} \end{array}} \right]$$

which is given in Supplementary Table S1. Once we find the genotypic frequencies using Eq. (5), we can find the recombination fraction at generation *t* by

8 $$\theta_{t+1}=W^{T}G_{t+1}=W^{T}P^{t}G_1$$

where $W$ is a $16 \times 1$ vector of weights that are given by the last column of Table 3. As the number of generations increases, the recombination fraction reaches its limit,

9 $$\mathop {\lim }\limits_{t \to \infty } {\theta_{t + 1}} = \mathop {\lim }\limits_{t \to \infty } {W^T}{P^t}{G_1} = {\rho_{{\text{self}}}} = \frac{2\theta }{{1 + 2\theta }}$$

**Table 3:** Recombinant gamete probabilities from all 16 fully phased genotypes and the sum of the two columns used as weights

Entry	Genotype	Pr(*Ab*)	Pr(*aB*)	*W* = Pr(*Ab*) + Pr(*aB*)
1	*AB/AB*	0	0	0
2	*AB/Ab*	1/2	0	1/2
3	*AB/aB*	0	1/2	1/2
4	*AB/ab*	$\tfrac{1}{2}\theta$	$\tfrac{1}{2}\theta$	$\theta$
5	*Ab/AB*	1/2	0	1/2
6	*Ab/Ab*	1	0	1
7	*Ab/aB*	$\tfrac{1}{2}(1 - \theta )$	$\tfrac{1}{2}(1 - \theta )$	$1 - \theta$
8	*Ab/ab*	1/2	0	1/2
9	*aB/AB*	0	1/2	1/2
10	*aB/Ab*	$\tfrac{1}{2}(1 - \theta )$	$\tfrac{1}{2}(1 - \theta )$	$1 - \theta$
11	*aB/aB*	0	1	1
12	*aB/ab*	0	1/2	1/2
13	*ab/AB*	$\tfrac{1}{2}\theta$	$\tfrac{1}{2}\theta$	$\theta$
14	*ab/Ab*	1/2	0	1/2
15	*ab/aB*	0	1/2	1/2
16	*ab/ab*	0	0	0

For example, when $\theta = 0.1$ in the F_2_ population, the final recombination fraction in the limit is

10 $${\rho_{{\text{self}}}} = \frac{2\theta }{{1 + 2\theta }} = \frac{2 \times 0.1}{{1 + 2 \times 0.1}} = \frac{1}{6} = 0.166667$$

Robbins [16] pooled the 16 fully phased genotypes into 10 genotypes and developed a $10 \times 10$ transition matrix. His approach was presented in Supplementary Note S1 for completeness of the study. Haldane and Waddington [6] further pooled the genotypes into five classes and developed a $5 \times 5$ transition matrix. Their result is presented in Supplementary Note S2.

### Recurrent equations for brother-sister mating

Recombinant inbred lines generated from brother sister mating is much more complicated than from self-fertilization. Haldane and Waddington [6] provided the recurrent equations of genotypes and derived the asymptotic solution for the recombination fraction when $t = \infty$, which is

11 $${\rho_{{\text{sib}}}} = \frac{4\theta }{{1 + 6\theta }}$$

Each sibling can take one of the 16 possible fully phased genotypes. Therefore, a sib pair can have a total of $16 \times 16 = 256$ genotype combinations. If we ignore the phase information, there are 10 possible genotypes per individual [16], a sib pair can take one of the $10 \times 10 = 100$ possible genotypes. Haldane and Waddington [6] pooled the 100 genotypes into 22 composite genotypes and developed recurrent equations for the 22 composite genotypes at generation $t + 1$ from the frequencies at generation *t*.

We now take advantage of the computer program to generate the $256 \times 256$ transition probability matrix and calculate the frequencies of the 256 pairs of genotypes of the sib-pairs. To build the recurrent equations, we first need to arrange the 16 possible genotypes of the first sib in the same way as shown in Table 2. We now nest the second sib’s 16 genotypes within each of the first sib. After defining the order of the sib-pair genotypes in ${G_t}$ (a $256 \times 1$ vector), we are ready to define the transition probability table $P$ (a $256 \times 256$ matrix). Recall that the last four columns of Table 2 form a $16 \times 4$ $H$ matrix. This matrix is also the basic element to develop the transition probability matrix. First, we need to define the sib pair in the position of vector ${G_t}$. If the first sib is entry *i* and the second sib is entry *j*, for $i,j = 1, \cdots ,16$, the corresponding sib pair position in vector ${G_t}$ is defined as

12 $$k = (i - 1) \times 16 + j$$

for $k = 1, \cdots ,256$ and $i,j = 1, \cdots ,16$. For example, when $i = 4$, $j = 10$, the subscript of ${P_{k}}$ is

$$k = (i - 1) \times 16 + j = (4 - 1) \times 16 + 10 = 48 + 10 = 58$$

The *k*th column of matrix *P* is

13 $${P_{\cdot k}} = {\text{vec}}(h_j^T{h_i}) \otimes {\text{vec}}(h_j^T{h_i})$$

We now demonstrate the formation of a few columns of the transition matrix. First, let us demonstrate the second sib-pair, *AB/AB* vs. *AB/Ab*. The gamete probabilities of the sib pair are ${h_1}$ and ${h_2}$, respectively. Let us define

$$h_2^T{h_1} = \left[ {\begin{array}{*{20}{c}} {\tfrac{1}{2}} \\ {\tfrac{1}{2}} \\ 0 \\ 0 \end{array}} \right]\left[ {\begin{array}{*{20}{c}} 1&0&0&0 \end{array}} \right] = \left[ {\begin{array}{*{20}{c}} {\tfrac{1}{2}}&0&0&0 \\ {\tfrac{1}{2}}&0&0&0 \\ 0&0&0&0 \\ 0&0&0&0 \end{array}} \right]$$

Therefore, the vectorization of $h_j^T{h_i}$ is

$${\text{vec}}(h_2^T{h_1}) = {\left[ {\begin{array}{*{20}{c}} {\tfrac{1}{2}}&{\tfrac{1}{2}}&0&0&0&0&0&0&0&0&0&0&0&0&0&0 \end{array}} \right]^T}$$

The column of the transition matrix corresponding to $i = 1$ and $j = 2$ is

$$k = (i - 1) \times 16 + j = (1 - 1) \times 16 + 2 = 2$$

Therefore, the 2nd column of matrix *P* is

14 $${P_{\cdot2}} = {\text{vec}}(h_2^T{h_1}) \otimes {\text{vec}}(h_2^T{h_1})$$

Let us now illustrate another sib pair, *AB/ab* vs. *Ab/aB*, where the first sib corresponds to entry number $i = 4$ and the second sib corresponds to entry number $j = 10$. The sib-pair corresponds to column number

$$k = (i - 1) \times 16 + j = (4 - 1) \times 16 + 10 = 58$$

of the transition matrix. We first define

$$h_{10}^T{h_4} = \left[ {\begin{array}{*{20}{c}} {\tfrac{1}{2}\theta } \\ {\tfrac{1}{2}(1 - \theta )} \\ {\tfrac{1}{2}(1 - \theta )} \\ {\tfrac{1}{2}\theta } \end{array}} \right]\left[ {\begin{array}{*{20}{c}} {\tfrac{1}{2}(1 - \theta )}&{\tfrac{1}{2}\theta }&{\tfrac{1}{2}\theta }&{\tfrac{1}{2}(1 - \theta )} \end{array}} \right] = \left[ {\begin{array}{*{20}{c}} {\tfrac{1}{4}\theta (1 - \theta )}&{\tfrac{1}{4}{\theta^2}}&{\tfrac{1}{4}{\theta^2}}&{\tfrac{1}{4}\theta (1 - \theta )} \\ {\tfrac{1}{4}{{(1 - \theta )}^2}}&{\tfrac{1}{4}\theta (1 - \theta )}&{\tfrac{1}{4}\theta (1 - \theta )}&{\tfrac{1}{4}{{(1 - \theta )}^2}} \\ {\tfrac{1}{4}{{(1 - \theta )}^2}}&{\tfrac{1}{4}\theta (1 - \theta )}&{\tfrac{1}{4}\theta (1 - \theta )}&{\tfrac{1}{4}{{(1 - \theta )}^2}} \\ {\tfrac{1}{4}\theta (1 - \theta )}&{\tfrac{1}{4}{\theta^2}}&{\tfrac{1}{4}{\theta^2}}&{\tfrac{1}{4}\theta (1 - \theta )} \end{array}} \right]$$

We then form the 58th column vector of matrix *P*,

$${P_{\cdot58}} = {\text{vec}}(h_{10}^T{h_4}) \otimes {\text{vec}}(h_{10}^T{h_4})$$

We start from the first column of matrix *P* to the last column of *P* to complete all 256 columns of the *P* matrix, i.e.,

$$P = \left[ {\begin{array}{*{20}{c}} {{P_{\cdot1}}}&{{P_{\cdot2}}}& \cdots &{{P\cdot_{256}}} \end{array}} \right]$$

The frequencies of the 256 sib pair genotypes at generation *t* are then used to calculate the frequencies of the sib pair combination for generation $t + 1$, as shown below,

15 $${G_{t + 1}} = P{G_t} = {P^t}{G_1}$$

How do we determine the initial sib-pair frequencies? Assume that the initial population is the ${{\text{F}}_1}$ hybrid, which represents entries of $i = 4$ ($AB/ab$) and $j = 13$ ($ab/AB$) as shown in Table 2. Therefore, the corresponding sib pairs among all $16 \times 16 = 256$ sib-pairs with both sibs being ${{\text{F}}_1}$ hybrids are identified as the following four entries,

$$\begin{gathered} {k_1} = (i - 1) \times 16 + i = (4 - 1) \times 16 + 4 = 52 \hfill \\ {k_2} = (i - 1) \times 16 + j = (4 - 1) \times 16 + 13 = 61 \hfill \\ {k_3} = (j - 1) \times 16 + i = (13 - 1) \times 16 + 4 = 196 \hfill \\ {k_4} = (j - 1) \times 16 + j = (13 - 1) \times 16 + 13 = 205 \hfill \\ \end{gathered}$$

Therefore, the initial sib-pairs frequencies are

$${G_1}[52] = {G_1}[61] = {G_1}[196] = {G_1}[205] = 1/4$$

and

$$G_1[k]=0,\forall{k}\not\ni{k_1},\,k_2,\,k_3,\,k_4$$

Recall that the last column of Table 3 forms a $16 \times 1$ weight vector denoted by $W$. We now build two vectors from vector *W*. The first one is

$${V_1} = W \otimes {J_{16 \times 1}}$$

and the second one is

$${V_2} = {J_{16 \times 1}} \otimes W$$

where ${J_{16 \times 1}}$ is a $16 \times 1$ unity vector (all 16 elements are ones) and $X \otimes Y$ is the Kronecker product between matrices *X* and *Y*. The final weight vector is the average of the two, i.e.,

16 $$V = \frac{1}{2}({V_1} + {V_2})$$

which forms a new $256 \times 1$ vector of weights to calculate the recombination fraction at generation $t + 1$.

17 $${\theta_{t + 1}} = {V^T}{G_{t + 1}} = {V^T}{P^t}{G_1}$$

As the number of generations of sib-mating increases, the recombination fraction reaches its limit,

18 $$\mathop {\lim }\limits_{t \to \infty } {\theta_{t + 1}} = \mathop {\lim }\limits_{t \to \infty } {V^T}{P^t}{G_1} = {\rho_{{\text{sib}}}} = \frac{4\theta }{{1 + 6\theta }}$$

For example, if $\theta = 0.1$, the final recombination fraction in the limit is

19 $${\rho_{{\text{sib}}}} = \frac{4\theta }{{1 + 6\theta }} = \frac{4 \times 0.1}{{1 + 6 \times 0.1}} = \frac{1}{4} = 0.25$$

### Recurrent equations of gametic frequencies in random mating

Random mating occurs starting from the ${{\text{F}}_1}$ hybrid to generate the ${{\text{F}}_2}$ and subsequent generations. Such a population is called the advanced intercross lines (AIL) by Darvasi and Soller [4]. The advantage of AILs is that linkage between tightly linked loci can be broken thereby increasing recombination. This results in a so-called expanded genetic map where estimated distances appear larger than those of the initial intercross. Such a particular design is useful to order genes or markers in strong linkage at the same locus. For instance, AILs were used for fine mapping in plant genetics [1] and animal genetics [11]. When $t \to \infty$, the recombination fraction reaches the limit, that is 1/2. Therefore, QTL mapping can be done when $t$ is not too large. There are several different ways to derive the recurrent equations for the recombination fraction. Robbins’ [16] derivation is general so that the initial genotype can be any of the 16 possible genotypes while the derivation of Darvasi and Soller [4] is simple but only applies to the initial genotype of *AB/ab*. The recombination fraction at $t$ can be expressed as a function of the recombination fraction at the F_2_ generation.

20 $${\theta_t} = \frac{1}{2}\left[ {1 - (1 - 2\theta ){{(1 - \theta )}^{t - 2}}} \right]$$

for $t \geqslant 2$. One can verify that when $t = 2$, ${\theta_2} = \theta$, which is indeed the recombination fraction of the ${{\text{F}}_2}$ population. Denote the four gametic frequencies by a row vector,

21 $$G = \left[ {\begin{array}{*{20}{c}} p&q&s&t \end{array}} \right]$$

where $p = \Pr (AB)$, $q = \Pr (Ab)$, $s = \Pr (aB)$, and $t=\text{Pr}(ab)$. Let

22 $${G_t} = \left[ {\begin{array}{*{20}{c}} {p_t}&{q_t}&{s_t}&{t_t} \end{array}} \right]$$

be a $1 \times 4$ frequency vector of the four gametes at generation *t*. The recurrent equations of the gametic frequencies are

23 $${G_{t + 1}} = ({G_t} \otimes {G_t})H$$

where *H* is the $16 \times 4$ matrix given in Table 2. For the F_2_ population, the four gametic frequencies are

24 $${G_1} = \left[ {\begin{array}{*{20}{c}} {\tfrac{1}{2}(1 - \theta )}&{\tfrac{1}{2}\theta }&{\tfrac{1}{2}\theta }&{\tfrac{1}{2}(1 - \theta )} \end{array}} \right]$$

In contrast to the previous mating systems, the gametic frequencies at generation $t + 1$ for random mating are not linear functions of the gametic frequencies at generation *t*. The recombination fraction for generation *t* is

25 $${\theta_t} = {G_t}[2] + {G_t}[3] = {q_t} + {s_t}$$

If the initial gametic frequency vector is the one given in Eq. (24), the limit of ${\theta_t}$ is 0.5 as $t \to \infty$. Using the result of Robbins [16], we can prove Eq. (20), which is presented in Supplementary Note S3.

## Results

### Self fertilization

**Fig. 1:**
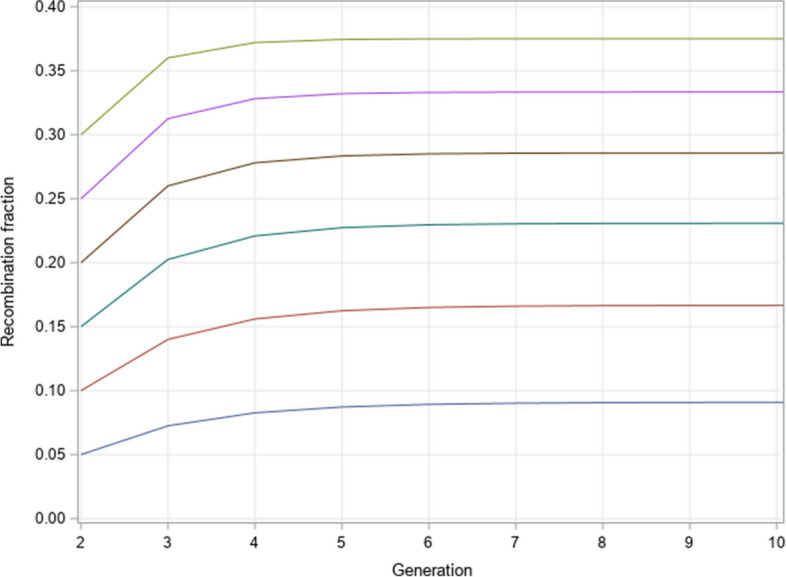
Recombination fraction profiles after 10 generations of self-fertilization

We first demonstrate the recombination fraction trajectory across generations when self-fertilization starts from the F_1_ hybrid, i.e., the initial genotype frequencies for AB/ab and ab/AB are 1/2 and 1/2, and 0 for all other genotypes. The initial recombination fraction was set at the following levels: 0.05, 0.1, 0.15, 0.2, 0.25 and 0.3. We evaluate the trajectory of recombination fractions for 10 generations, as shown in Fig. 1. After 10 generations of self-fertilization, they all reach their asymptotic values, which are presented in Table 4. For example, when the initial recombination fraction is $\theta = 0.20$, the asymptotic recombination fraction is

26 $${\rho_{Self}} = \frac{2\theta }{{1 + 2\theta }} = \frac{2 \times 0.20}{{1 + 2 \times 0.20}} = 0.285714$$

**Table 4:** Asymptotic recombination fractions from different initial recombination fractions after repeated self-fertilization for 10 generations

Initial recombination fraction ($\theta$)	Asymptotic recombination fraction ($\rho$)
0.05	0.090909
0.10	0.166667
0.15	0.230769
0.20	0.285714
0.25	0.333333
0.30	0.375000

### Brother-sister mating

Among all $16 \times 16 = 256$ sib-pairs with both sibs being ${{\text{F}}_1}$ hybrids are identified as the following four entries,

$$\begin{gathered} {k_1} = (i - 1) \times 16 + i = (4 - 1) \times 16 + 4 = 52 \hfill \\ {k_2} = (i - 1) \times 16 + j = (4 - 1) \times 16 + 13 = 61 \hfill \\ {k_3} = (j - 1) \times 16 + i = (13 - 1) \times 16 + 4 = 196 \hfill \\ {k_4} = (j - 1) \times 16 + j = (13 - 1) \times 16 + 13 = 205 \hfill \\ \end{gathered}$$

Therefore, the initial frequencies are

$${G_0}[52] = {G_0}[61] = {G_0}[196] = {G_0}[205] = 1/4$$

and

$${G_0}[k] = 0,\forall k \notin {k_1},{k_2},{k_3},{k_4}$$

**Fig. 2:**
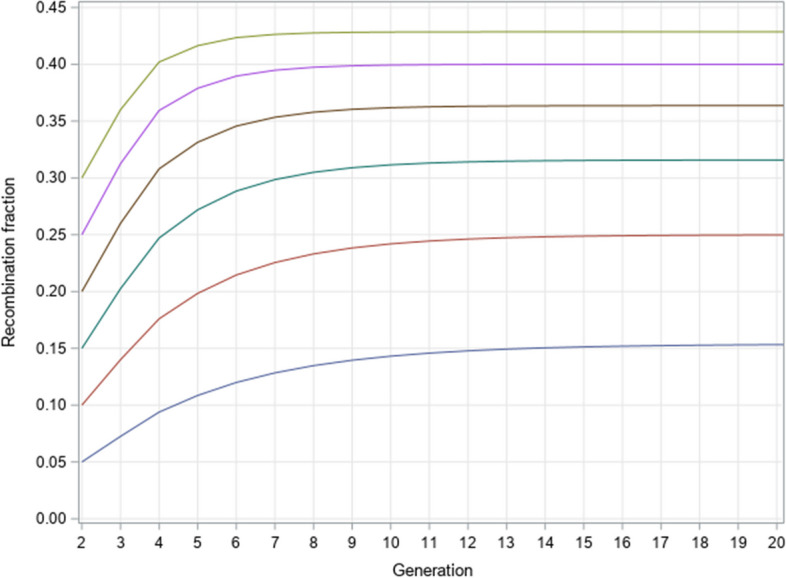
Recombination fraction profiles after 20 generations of brother-sister mating

Again, we demonstrate the recombination fraction profiles across generations for brother-sister mating starting from the F_1_ population. The four sib pairs corresponding to the double heterozygote are (AB/ab-AB/ab), (AB/ab-ab/AB), (ab/AB-AB/ab) and (ab/AB-ab/AB). The initial recombination fraction was set at the following levels: 0.05, 0.1, 0.15, 0.2, 0.25 and 0.3. We evaluated the recombination fractions change for 20 generations. Figure 2 shows the recombination fraction trajectories. After 20 generations of brother-sister mating, they all reach their equilibrium values, which are presented in Table 5. For example, when the initial recombination fraction is $\theta = 0.20$, the asymptotic recombination fraction is

27 $${\rho_{Sib}} = \frac{4\theta }{{1 + 6\theta }} = \frac{4 \times 0.20}{{1 + 6 \times 0.20}} = 0.3636364$$

**Table 5:** Asymptotic recombination fractions from different initial recombination fractions after repeated brother-sister mating for 20 generation

Initial recombination fraction ($\theta$)	Asymptotic recombination fraction ($\rho$)
0.05	0.1538462
0.10	0.2500000
0.15	0.3157895
0.20	0.3636364
0.25	0.4000000
0.30	0.4285714

### Random mating

**Fig. 3:**
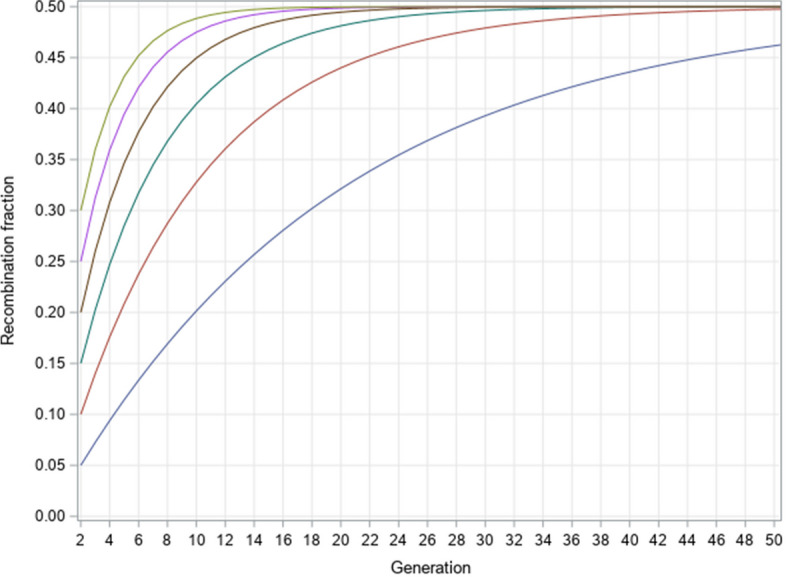
Recombination fraction profiles after 50 generations of random mating

Starting from the F_1_ hybrid, the population went to 50 generations of random mating. The recombination fraction profiles are demonstrated in Fig. 3 from various initial values of the recombination fraction.

After 50 generations of random mating, all populations have reached their equilibrium value (${\rho_{Random}} = 0.5$) except that the population starting with $\theta = 0.05$ has not reached the equilibrium, but with a recombination fraction of 0.4653748 at $t = 50$, which is calculated via

28 $${\theta_{50}} = \frac{1}{2}\left[ {1 - (1 - 2\theta ){{(1 - \theta )}^{50 - 2}}} \right] = \frac{1}{2}\left[ {1 - (1 - 2 \times 0.05){{(1 - 0.05)}^{48}}} \right] = 0.4616$$

Three R functions were provided to calculate the recombination fractions for PRERILs developed via self-fertilization, brother-sister mating and random mating (Supplementary Code S1).

### Comparison of the three mating systems

**Fig. 4:**
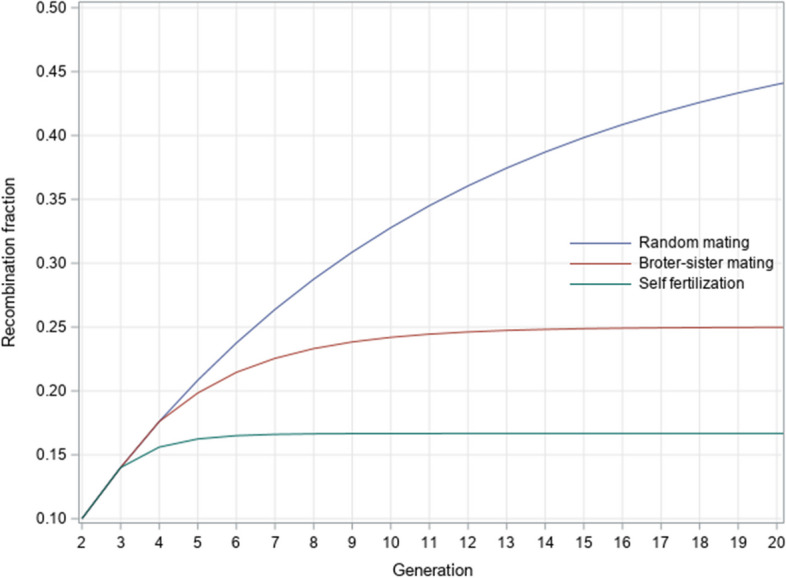
Comparison of recombination fraction profiles among three mating systems

Starting with the same recombination fraction of 0.10, all three mating systems (self-fertilization, brother-sister mating, and random mating) underwent 20 consecutive generations of reproduction. The trajectories of the recombination fraction are compared among the three mating systems (Fig. 4).

At generation 2 and 3, all three mating systems have the same recombination fraction. Self-fertilization starts splitting from the other two systems after generation 3 while brother-sister mating deviates from random mating after generation 4. Four generations of self-fertilization are technically sufficient to make the recombination fraction reach the asymptotic value. Ten generations of brother-sister mating are sufficient to make the recombination fraction reach its equilibrium value.

### Validation of the recurrent equations via Monte Carlo simulations

The recurrent equations derived here must be correct because the asymptotic results ($t > 10$ for self-fertilization and $t > 20$ for brother-sister mating) match the final results provided by Haldane and Waddington [6] and Robbins [14]. To doubly ensure the correctness of the derivation, we decided to further validate the recurrent equations via Monte Carlo simulations. The assumptions required to derive the recurrent equations are (1) there is no interference in crossovers between two genomic segments [5]; (2) there is no segregation distortion of the markers under investigation. As a result, the best way to validate the recurrent equations is to simulate RILs based on these assumptions. It is hard to use actual data from RILs for validation because these two assumptions may not be satisfied in reality. Another justification for using simulations to validate the derivation is that the derivation is based on expectations of the genotype groups and the expectations only apply to large samples, in fact, infinitely large samples. In reality, the sample sizes of real populations are always finite. The theoretical derivation cannot be validated based on one or a few finite samples.

**Fig. 5:**
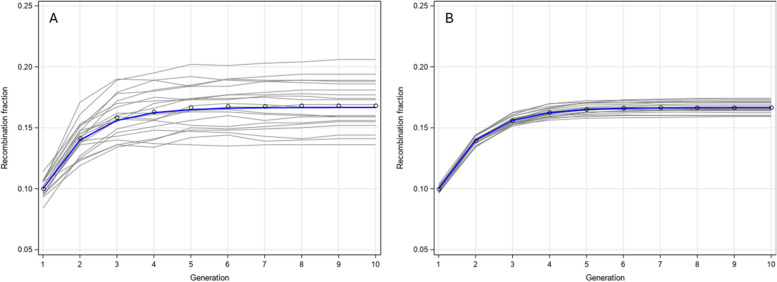
Simulation results of self-fertilization for 10 generations. Panel **A** shows 20 replications of sample size 500. Panel **B** shows 20 replications of sample size 10,000. The blue curves are the theoretical values from the recurrent equations while the scatter plots show the averages of the 20 replications

The recurrent equation of the recombination fraction for random matting was originally derived by Jennings [9] and later by Darvasi and Soller [4]. Our derivation is merely an alternative approach to obtain the same result. Therefore, no validation is needed for random mating. To validate the recurrent equations for self-fertilization, we started with a single F1 individual with genotype AB/ab. We replaced this phased genotype by 11/00, where 11 is the paternal haplotype and 00 is the maternal haplotype in the F1 founder. The distance between the two loci set at

29 $$\mu = - \frac{1}{2}\ln (1 - 2\theta) = - \frac{1}{2}\ln (1 - 2 \times 0.1) = {0}{\text{.1115718 Morgan}}$$

where the recombination fraction was set at $\theta= 0.1$. The simulation started at F2 from the F1 gametes. There were two random numbers involved in generating each gamete. The first random number was Bernoulli ${\delta_1}\sim {\text{Bernoulli}}(0.5)$, which determines the first allele of the gamete. If ${\delta_1} = 1$ then the gamete took 1 from the paternal allele; otherwise, the gamete took 0 from the maternal allele. Let us assume that ${\delta_1} = 1$ so that the paternal allele has passed to the gamete for the first locus. We then generated a Poisson random from mean $\mu = 0.1115718$, say $x = 0,1, \cdots ,\infty$, i.e., $x \sim {\text{Poisson}}(\mu )$. If $x$ is an odd number, then recombination has occurred and the second locus of the paternal allele has passed to the gamete, i.e., 0. If $x$ is an even number, recombination would not happen and thus the second locus of the gamete would remain 1 from the paternal allele. The same algorithm also applied to the maternal haplotype of the gamete. The two gametes merged together to make the genotype of the individual. This process continued until $t = 10$ generations. We generated 500 individuals from the single seed descent process to make up one RIL population. The recombination fraction at generation *t* was the proportion of the recombinant haplotypes, 10 or 01. Note that the two recombinant haplotypes are referred to the F_2_ generation. In later generations, 11 and 00 may be the recombinants. Eventually, we generated 20 populations, each consisting of 500 individuals. Figure 5 (panel A) shows the recombination fractions of each population against the generation index up to 10. Variation among the 20 replicates is very obvious. The average of the 20 replicates is shown in the scatter plot, which partially overlaps with theoretical curve in blue. Figure 5 (panel B) shows the same plots but the sample size of each population has been increased to 10,000. The average of the 20 populations (scatter plots) completely overlaps with the blue theoretical value. Even though the sample size was as large as 10,000, there were still variation among the replicates.

**Fig. 6:**
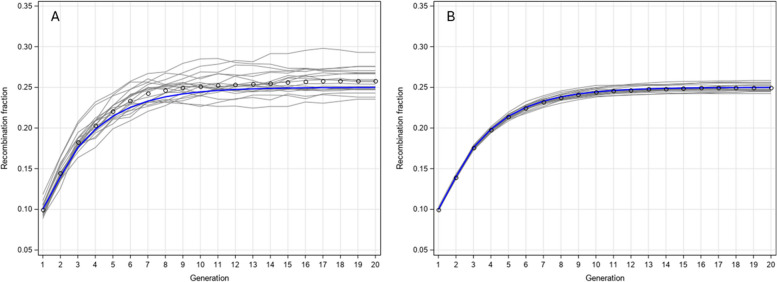
Simulation results of brother-sister mating for 20 generations. Panel **A** shows 20 replications for sample size of 500 mating pairs. Panel **B** shows 20 replications for sample size of 10,000 mating pairs. The blue curves are the theoretical values from the recurrent equations while the scatter plots show the averages of the 20 replications

We also simulated brother-sister mating for 20 generations with sample size of 500 mating pairs and 10,000 mating pairs, respectively. Both schemes were replicated 20 times. The results are shown in Fig. 6, where Fig. 6A shows the plots for sample size of 500 mating pairs and Fig. 6B shows the results for sample size of 10,000 mating pairs. The variation among the 20 replicates was smaller than the variation in self-fertilization because the sample size was actually doubled in brother-sister mating. The simulation studies have successfully validated the theoretical derivation of the recurrent equations.

### Incorporation of the modified recombination fraction in QTL mapping

Starting with $\theta = 0.05$, if the F_1_ hybrid undergoes 3 generations of self-fertilization, the recombination fraction will change from $\theta = 0.05$ in F_2_ to ${\theta_4} = 0.08923$ in F_4_. The heterozygosity will be reduced from 0.5 to

30 $${H_4} = {\left( \frac{1}{2} \right)^{4 - 1}} = \frac{1}{8} = 0.125$$

QTL mapping using ${F_4}$ as the source population requires a new approach to calculate the conditional probabilities of QTL genotypes given flanking marker genotypes. This is due to (1) The recombination fraction between two loci has been modified from $\theta$ in the initial population to ${\theta_4}$ in the F_4_ population; (2) The heterozygosity has been modified from ${H_2} = 0.50$ to ${H_4} = 0.125$. Below is an example showing the differences in QTL mapping between F_2_ and F_4_.

#### QTL mapping in F_2_ populations

For the F_2_ population, let the recombination fraction between the two flanking markers (A and B) be ${\theta_{AB}} = 0.05$, the recombination fraction between A and Q be ${\theta_{AQ}} = 0.01$ and thus the recombination fraction between Q and B is

31 $${\theta_{QB}} = ({\theta_{AB}} - {\theta_{AQ}})/(1 - 2{\theta_{AQ}}) = (0.05 - 0.01)/(1 - 2 \times 0.01) = 0.0408$$

where the order of the three loci is A-Q-B. Let us denote the genotypes of the three loci by

$$A = \left\{ {\begin{array}{*{20}{c}} 1 \\ 2 \\ 3 \end{array}} \right.\begin{array}{*{20}{c}} {} \\ {} \\ {} \end{array}\begin{array}{*{20}{c}} {{\text{for}}} \\ {{\text{for}}} \\ {{\text{for}}} \end{array}\begin{array}{*{20}{c}} {AA} \\ {Aa} \\ {aa} \end{array}\begin{array}{*{20}{c}} {} \\ {} \\ {} \end{array}\begin{array}{*{20}{c}} {\Pr (AA) = 0.25} \\ {\Pr (Aa) = 0.50} \\ {\Pr (aa) = 0.25} \end{array};B = \left\{ {\begin{array}{*{20}{c}} 1 \\ 2 \\ 3 \end{array}} \right.\begin{array}{*{20}{c}} {} \\ {} \\ {} \end{array}\begin{array}{*{20}{c}} {{\text{for}}} \\ {{\text{for}}} \\ {{\text{for}}} \end{array}\begin{array}{*{20}{c}} {BB} \\ {Bb} \\ {bb} \end{array}\begin{array}{*{20}{c}} {} \\ {} \\ {} \end{array}\begin{array}{*{20}{c}} {\Pr (BB) = 0.25} \\ {\Pr (Bb) = 0.50} \\ {\Pr (bb) = 0.25} \end{array};Q = \left\{ {\begin{array}{*{20}{c}} 1 \\ 2 \\ 3 \end{array}} \right.\begin{array}{*{20}{c}} {} \\ {} \\ {} \end{array}\begin{array}{*{20}{c}} {{\text{for}}} \\ {{\text{for}}} \\ {{\text{for}}} \end{array}\begin{array}{*{20}{c}} {QQ} \\ {Qq} \\ {qq} \end{array}\begin{array}{*{20}{c}} {} \\ {} \\ {} \end{array}\begin{array}{*{20}{c}} {\Pr (QQ) = 0.25} \\ {\Pr (Qq) = 0.50} \\ {\Pr (qq) = 0.25} \end{array}$$

The conditional probabilities of the QTL genotype given the flanking marker genotypes is defined from the following Bayes theorem,

32 $$\Pr (Q = k|A = i,B = j) = \frac{\Pr (Q = k)\Pr (A = i|Q = k)\Pr (B = j|Q = k)}{{\sum\nolimits_{k^{\prime} = 1}^3 {\Pr (Q = k^{\prime})\Pr (A = i|Q = k^{\prime})\Pr (B = j|Q = k^{\prime})} }}$$

where $\Pr (Q = k)$ is the marginal probability of the QTL genotype, $\Pr (A = i|Q = k)$ and $\Pr (B = j|Q = k)$ are the conditional probabilities of the marker genotypes given the QTL genotype. The conditional probabilities are extracted from the following two transition matrices,

$$\begin{aligned} {T_{A/Q}} = & \left[ {\begin{array}{*{20}{c}} {\Pr (A = 1|Q = 1)}&{\Pr (A = 2|Q = 1)}&{\Pr (A = 3|Q = 1)} \\ {\Pr (A = 1|Q = 2)}&{\Pr (A = 2|Q = 2)}&{\Pr (A = 3|Q = 2)} \\ {\Pr (A = 1|Q = 3)}&{\Pr (A = 2|Q = 3)}&{\Pr (A = 3|Q = 3)} \end{array}} \right] \\ = & \left[ {\begin{array}{*{20}{c}} {{{(1 - {\theta_{QA}})}^2}}&{2(1 - {\theta_{QA}}){\theta_{QA}}}&{\theta_{QA}^2} \\ {(1 - {\theta_{QA}}){\theta_{QA}}}&{{{(1 - {\theta_{QA}})}^2} + \theta_{QA}^2}&{(1 - {\theta_{QA}}){\theta_{QA}}} \\ {\theta_{QA}^2}&{2(1 - {\theta_{QA}}){\theta_{QA}}}&{{{(1 - {\theta_{QA}})}^2}} \end{array}} \right] \\ & \\ \end{aligned}$$

and

$$\begin{aligned} {T_{B/Q}} = & \left[ {\begin{array}{*{20}{c}} {\Pr (B = 1|Q = 1)}&{\Pr (B = 2|Q = 1)}&{\Pr (B = 3|Q = 1)} \\ {\Pr (B = 1|Q = 2)}&{\Pr (B = 2|Q = 2)}&{\Pr (B = 3|Q = 2)} \\ {\Pr (B = 1|Q = 3)}&{\Pr (B = 2|Q = 3)}&{\Pr (B = 3|Q = 3)} \end{array}} \right] \\ = & \left[ {\begin{array}{*{20}{c}} {{{(1 - {\theta_{QB}})}^2}}&{2(1 - {\theta_{QB}}){\theta_{QB}}}&{\theta_{QB}^2} \\ {(1 - {\theta_{QB}}){\theta_{QB}}}&{{{(1 - {\theta_{QB}})}^2} + \theta_{QB}^2}&{(1 - {\theta_{QB}}){\theta_{QB}}} \\ {\theta_{QB}^2}&{2(1 - {\theta_{QB}}){\theta_{QB}}}&{{{(1 - {\theta_{QB}})}^2}} \end{array}} \right] \\ \end{aligned}$$

For example, the conditional probability that the QTL genotype is Qq given the genotype of locus A is AA and the genotype of locus B is Bb is

33 $$\Pr (Q = 2|A = 1,B = 2) = \frac{\Pr (Q = 2)\Pr (A = 1|Q = 2)\Pr (B = 2|Q = 2)}{{\sum\nolimits_{k^{\prime} = 1}^3 {\Pr (Q = k^{\prime})\Pr (A = 1|Q = k^{\prime})\Pr (B = 2|Q = k^{\prime})} }}$$

where

34 $$\text{Pr}(Q=1)\text{Pr}(A=1|Q=1)\text{Pr}(B=2|Q=1)=\frac{1}{4}(1-\theta_{QA})^2\times2\theta_{QB}(1-\theta_{QB})=0.0191856$$

35 $$\Pr (Q = 2)\Pr (A = 1|Q = 2)\Pr (B = 2|Q = 2) = \frac{1}{2}(1 - {\theta_{QA}}){\theta_{QA}}\left[ {{{(1 - {\theta_{QB}})}^2} + \theta_{QB}^2} \right] = 0.0045624$$

36 $$\Pr (Q = 3)\Pr (A = 1|Q = 3)\Pr (B = 2|Q = 3) = \frac{1}{4}\theta_{QA}^2 \times 2{\theta_{QB}}(1 - {\theta_{QB}}) = 1.9575{\text{E}} - 6$$

The denominator is the sum of the three terms,

$$\sum\nolimits_{k^{\prime} = 1}^3 {\Pr (Q = k^{\prime})\Pr (A = 1|Q = k^{\prime})\Pr (B = 2|Q = k^{\prime})} = \frac{1}{2}{\theta_{AB}}(1 - {\theta_{AB}}) = 0.02375$$

Therefore, the three conditional probabilities of the QTL genotypes are

37 $$\Pr (Q = 1|A = 1,B = 2) = \frac{{{{(1 - {\theta_{QA}})}^2}{\theta_{QB}}(1 - {\theta_{QB}})}}{{{\theta_{AB}}(1 - {\theta_{AB}})}} = 0.807816$$

38 $$\Pr (Q = 2|A = 1,B = 2) = \frac{{(1 - {\theta_{QA}}){\theta_{QA}}\left[ {{{(1 - {\theta_{QB}})}^2} + \theta_{QB}^2} \right]}}{{{\theta_{AB}}(1 - {\theta_{AB}})}} = 0.1921015$$

39 $$\Pr (Q = 3|A = 1,B = 2) = \frac{{\theta_{QA}^2{\theta_{QB}}(1 - {\theta_{QB}})}}{{{\theta_{AB}}(1 - {\theta_{AB}})}} = 0.0000824$$

Here is another example, the conditional probability that the QTL genotype is Qq given the genotype of locus A is AA and the genotype of locus B is BB is

40 $$\Pr (Q = 2|A = 1,B = 1) = \frac{\Pr (Q = 2)\Pr (A = 1|Q = 2)\Pr (B = 1|Q = 2)}{{\sum\nolimits_{k^{\prime} = 1}^3 {\Pr (Q = k^{\prime})\Pr (A = 1|Q = k^{\prime})\Pr (B = 1|Q = k^{\prime})} }}$$

where

41 $$\Pr (Q = 1)\Pr (A = 1|Q = 1)\Pr (B = 1|Q = 1) = \frac{1}{4}{(1 - {\theta_{QA}})^2}{(1 - {\theta_{QB}})^2}$$

42 $$\Pr (Q = 2)\Pr (A = 1|Q = 2)\Pr (B = 1|Q = 2) = \frac{1}{2}(1 - {\theta_{QA}}){\theta_{QA}}(1 - {\theta_{QB}}){\theta_{QB}}$$

43 $$\Pr (Q = 3)\Pr (A = 1|Q = 3)\Pr (B = 1|Q = 3) = \frac{1}{4}\theta_{QB}^2\theta_{QA}^2$$

The denominator is the sum of the three terms,

44 $$\begin{gathered} \frac{1}{4}{(1 - {\theta_{QA}})^2}{(1 - {\theta_{QB}})^2} + \frac{1}{2}(1 - {\theta_{QA}}){\theta_{QA}}(1 - {\theta_{QB}}){\theta_{QB}} + \frac{1}{4}\theta_{QB}^2\theta_{QA}^2 \hfill \\ = \frac{1}{4}{\left[ {(1 - {\theta_{QA}})(1 - {\theta_{QB}}) + \theta_{QB}\theta_{QA}} \right]^2} \hfill \\ = \frac{1}{4}{\left[ {1 - ({\theta_{QB}} + {\theta_{QA}} - 2{\theta_{QA}}{\theta_{QB}})} \right]^2} \hfill \\ = \frac{1}{4}{(1 - {\theta_{AB}})^2} \hfill \\ \end{gathered}$$

Therefore, the three conditional probabilities of the QTL genotypes given the genotypes of markers A and B are

45 $$\Pr (Q = 1|A = 1,B = 1) = \frac{{\tfrac{1}{4}{{(1 - {\theta_{QA}})}^2}{{(1 - {\theta_{QB}})}^2}}}{{\tfrac{1}{4}{{(1 - {\theta_{AB}})}^2}}} = \frac{{{{(1 - {\theta_{QA}})}^2}{{(1 - {\theta_{QB}})}^2}}}{{{{(1 - {\theta_{AB}})}^2}}} = {0}{\text{.9991749}}$$

46 $$\begin{aligned} \Pr (Q = 2|A = 1,B = 1) = & \frac{{2(1 - {\theta_{QA}}){\theta_{QA}}(1 - {\theta_{QB}}){\theta_{QB}}}}{{{{(1 - {\theta_{AB}})}^2}}} \\ = & \frac{2 \times (1 - 0.01) \times 0.01 \times (1 - 0.0408) \times 0.0408}{{{{(1 - 0.05)}^2}}} \\ = & {0}{\text{.0008585929}} \\ \end{aligned}$$

and

47 $$\Pr (Q = 3|A = 1,B = 1) = \frac{{\tfrac{1}{4}\theta_{QA}^2\theta {{_{QB}^2}^2}}}{{\tfrac{1}{4}{{(1 - {\theta_{AB}})}^2}}} = \frac{{\theta_{QA}^2\theta {{_{QB}^2}^2}}}{{{{(1 - {\theta_{AB}})}^2}}} = {1}{\text{.844476E - 07}}$$

#### QTL mapping in F_4_ populations

For the F_4_ population, the recombination fraction between the two flanking markers (A and B) has changed from ${\theta_{AB}} = 0.05$ to $\theta_{AB}^{(4)} = 0.08923$, the recombination fraction between A and Q has changed from ${\theta_{AQ}} = 0.01$ to $\theta_{AQ}^{(4)} = 0.01905$ and thus the recombination fraction between Q and B in F_4_ is

48 $$\theta_{QB}^{(4)} = (\theta_{AB}^{(4)} - \theta_{AQ}^{(4)})/(1 - 2\theta_{AQ}^{(4)}) = (0.08923 - 0.01905)/(1 - 2 \times 0.01905) = 0.07296$$

where the order of the three loci is A-Q-B. Let us denote the genotypes of the three loci by

$$A = \left\{ {\begin{array}{*{20}{c}} 1 \\ 2 \\ 3 \end{array}} \right.\begin{array}{*{20}{c}} {} \\ {} \\ {} \end{array}\begin{array}{*{20}{c}} {{\text{for}}} \\ {{\text{for}}} \\ {{\text{for}}} \end{array}\begin{array}{*{20}{c}} {AA} \\ {Aa} \\ {aa} \end{array}\begin{array}{*{20}{c}} {} \\ {} \\ {} \end{array}\begin{array}{*{20}{c}} {\Pr (AA) = 0.4375} \\ {\Pr (Aa) = 0.125} \\ {\Pr (aa) = 0.4375} \end{array};B = \left\{ {\begin{array}{*{20}{c}} 1 \\ 2 \\ 3 \end{array}} \right.\begin{array}{*{20}{c}} {} \\ {} \\ {} \end{array}\begin{array}{*{20}{c}} {{\text{for}}} \\ {{\text{for}}} \\ {{\text{for}}} \end{array}\begin{array}{*{20}{c}} {BB} \\ {Bb} \\ {bb} \end{array}\begin{array}{*{20}{c}} {} \\ {} \\ {} \end{array}\begin{array}{*{20}{c}} {\Pr (BB) = 0.4375} \\ {\Pr (Bb) = 0.125} \\ {\Pr (bb) = 0.4375} \end{array};Q = \left\{ {\begin{array}{*{20}{c}} 1 \\ 2 \\ 3 \end{array}} \right.\begin{array}{*{20}{c}} {} \\ {} \\ {} \end{array}\begin{array}{*{20}{c}} {{\text{for}}} \\ {{\text{for}}} \\ {{\text{for}}} \end{array}\begin{array}{*{20}{c}} {QQ} \\ {Qq} \\ {qq} \end{array}\begin{array}{*{20}{c}} {} \\ {} \\ {} \end{array}\begin{array}{*{20}{c}} {\Pr (QQ) = 0.4375} \\ {\Pr (Qq) = 0.125} \\ {\Pr (qq) = 0.4375} \end{array}$$

Note that the superscript of $\theta$ is now the generation index because the subscript has been taken by the two loci. The conditional probabilities of the QTL genotype given the flanking marker genotypes is defined from the following Bayes theorem,

49 $$\Pr (Q = k|A = i,B = j) = \frac{\Pr (Q = k)\Pr (A = i|Q = k)\Pr (B = j|Q = k)}{{\sum\nolimits_{k^{\prime} = 1}^3 {\Pr (Q = k^{\prime})\Pr (A = i|Q = k^{\prime})\Pr (B = j|Q = k^{\prime})} }}$$

where $\Pr (Q = k)$ is the marginal probability of the QTL genotype, $\Pr (A = i|Q = k)$ and $\Pr (B = j|Q = k)$ are the conditional probabilities of the marker genotypes given the QTL genotype.

These transition probabilities are extracted from the following two transition matrices,

$${T_{A/Q}} = \left[ {\begin{array}{*{20}{c}} {{{(1 - \theta_{QA}^{(4)})}^2}}&{2(1 - \theta_{QA}^{(4)})\theta_{QA}^{(4)}}&{\theta_{QA}^{(4)2}} \\ {(1 - \theta_{QA}^{(4)})\theta_{QA}^{(4)}}&{{{(1 - \theta_{QA}^{(4)})}^2} + \theta_{QA}^{(4)2}}&{(1 - \theta_{QA}^{(4)})\theta_{QA}^{(4)}} \\ {\theta_{QA}^{(4)2}}&{2(1 - \theta_{QA}^{(4)})\theta_{QA}^{(4)}}&{{{(1 - \theta_{QA}^{(4)})}^2}} \end{array}} \right]$$

and

$${T_{B/Q}} = \left[ {\begin{array}{*{20}{c}} {{{(1 - \theta_{QB}^{(4)})}^2}}&{2(1 - \theta_{QB}^{(4)})\theta_{QB}^{(4)}}&{\theta_{QB}^{(4)2}} \\ {(1 - \theta_{QB}^{(4)})\theta_{QB}^{(4)}}&{{{(1 - \theta_{QB}^{(4)})}^2} + \theta_{QB}^{(4)2}}&{(1 - \theta_{QB}^{(4)})\theta_{QB}^{(4)}} \\ {\theta_{QB}^{(4)2}}&{2(1 - \theta_{QB}^{(4)})\theta_{QB}^{(4)}}&{{{(1 - \theta_{QB}^{(4)})}^2}} \end{array}} \right]$$

For example, the conditional probability that the QTL genotype is Qq given the genotype of locus A is AA and the genotype of locus B is Bb is

50 $$\Pr (Q = 2|A = 1,B = 2) = \frac{\Pr (Q = 2)\Pr (A = 1|Q = 2)\Pr (B = 2|Q = 2)}{{\sum\nolimits_{k^{\prime} = 1}^3 {\Pr (Q = k^{\prime})\Pr (A = 1|Q = k^{\prime})\Pr (B = 2|Q = k^{\prime})} }}$$

where

$$\begin{aligned} &\Pr (Q = 1) \Pr (A = 1|Q = 1)\Pr (B = 2|Q = 1) = 0.4375 \times {(1 - \theta_{QA}^{(4)})^2} \times 2\theta_{QB}^{(4)}(1 - \theta_{QB}^{(4)}) = 0.0569487 \\ \end{aligned}$$

$$\begin{aligned} \Pr (Q = 2) \Pr (A = 1|Q = 2)\Pr (B = 2|Q = 2) = 0.125 \times (1 - \theta_{QA}^{(4)})\theta_{QA}^{(4)}\left[ {{{(1 - \theta_{QB}^{(4)})}^2} + \theta_{QB}^{(4)2}} \right] = 0.0020199 \\ \end{aligned}$$

$$\begin{aligned} \Pr (Q = 3) \Pr (A = 1|Q = 3)\Pr (B = 2|Q = 3) = 0.4375 \times \theta_{QA}^{(4)2} \times 2\theta_{QB}^{(4)}(1 - \theta_{QB}^{(4)}) = 0.0000215 \\ \end{aligned}$$

The denominator is the sum of the three terms,

$$\sum\nolimits_{k^{\prime} = 1}^3 {\Pr (Q = k^{\prime})\Pr (A = 1|Q = k^{\prime})\Pr (B = 2|Q = k^{\prime})} = 0.0589901$$

Therefore, the three conditional probabilities of the QTL genotypes given the genotypes of markers A and B are

$$\Pr (Q = 1|A = 1,B = 2) = \frac{0.0569487}{{0.0589901}} = 0.9653945$$

$$\Pr (Q = 2|A = 1,B = 2) = \frac{0.0020199}{{0.0589901}} = 0.0342414$$

$$\Pr (Q = 3|A = 1,B = 2) = \frac{0.0000215}{{0.0589901}} = 0.0003641$$

The conditional probability that the QTL genotype is Qq given the genotype of locus A is AA and the genotype of locus B is BB is

$$\Pr (Q = 2|A = 1,B = 1) = \frac{\Pr (Q = 2)\Pr (A = 1|Q = 2)\Pr (B = 1|Q = 2)}{{\sum\nolimits_{k^{\prime} = 1}^3 {\Pr (Q = k^{\prime})\Pr (A = 1|Q = k^{\prime})\Pr (B = 1|Q = k^{\prime})} }}$$

where

$$\Pr (Q = 1)\Pr (A = 1|Q = 1)\Pr (B = 1|Q = 1) = 0.4375 \times {(1 - {\theta_{QA}})^2}{(1 - {\theta_{QB}})^2} = 0.3618003$$

$$\Pr (Q = 2)\Pr (A = 1|Q = 2)\Pr (B = 1|Q = 2) = 0.125 \times (1 - {\theta_{QA}}){\theta_{QA}}(1 - {\theta_{QB}}){\theta_{QB}} = 0.000158$$

$$\Pr (Q = 3)\Pr (A = 1|Q = 3)\Pr (B = 1|Q = 3) = 0.4375 \times \theta_{QB}^2\theta_{QA}^2 = 8.4515{\text{E}} - 7$$

The denominator is the sum of the three terms,

$$\sum\nolimits_{k^{\prime} = 1}^3 {\Pr (Q = k^{\prime})\Pr (A = 1|Q = k^{\prime})\Pr (B = 1|Q = k^{\prime})} = 0.3619592$$

Therefore, the three conditional probabilities of the QTL genotypes given the genotypes of markers A and B are

$$\Pr (Q = 1|A = 1,B = 1) = \frac{0.3618003}{{0.3619592}} = 0.9995612$$

$$\Pr (Q = 2|A = 1,B = 1) = \frac{0.000158}{{0.3619592}} = 0.0004365$$

and

$$\Pr (Q = 3|A = 1,B = 1) = \frac{{8.4515{\text{E}} - 7}}{0.3619592} = 2.3349{\text{E}} - 6$$

Table 6 summarizes the results of the conditional probabilities of the QTL genotypes given the flanking marker genotypes. Because of the heterozygosity reduction due to repeated inbreeding, the conditional probabilities of homozygotes in F_4_ are always higher than the homozygosity in F_2_.

**Table 6:** Comparison of the conditional probabilities of QTL genotypes given flanking marker genotypes between F_2_ and F_4_ generations of self-fertilization

	Prior		Posterior	
	F_2_	F_4_	F_2_	F_4_
Pr(Q = 1|A = 1,B = 2)	0.25	0.4375	0.807816	0.9653945
Pr(Q = 2|A = 1,B = 2)	0.5	0.125	0.1921015	0.0342414
Pr(Q = 3|A = 1,B = 2)	0.25	0.4375	0.0000824	0.0003641
Pr(Q = 1|A = 1,B = 1)	0.25	0.4375	0.9991409	0.9995612
Pr(Q = 2|A = 1,B = 1)	0.5	0.125	0.0008589	0.0004365
Pr(Q = 3|A = 1,B = 1)	0.25	0.4375	1.846E-7	2.3349E-6

### Validation from pre-recombinant inbred lines of rice

Xu et al. [18] generated 191 F_2_ plants from an elite hybrid rice (Shanyou63) derived from the cross of Zhenshan97 and Minghui63. From the 191 F_2_ plants, they further developed 191 F_3_ and 191 F_4_ plants by single seed descent. Genotypes of a total of 1696 marker bins were inferred from the DNA sequences for each plant of the three filiations. The data set was used to validate the Markov model. Technically, one pair of markers suffice to validate the model. We used markers of the 12th chromosome for validation. This is the shortest chromosome with 63 markers, all of which follow Mendelian segregations in all three filiations (F_2_, F_3_ and F_4_). The Mendelian ratio for F_2_ is $1:2:1$, for F_3_ is $3:2:3$ and for F_4_ is $7:2:7$, which were used as the theoretical proportions in the segregation distortion Chi-square tests. The 63 markers form $63 \times 62/2 = 1953$ marker pairs for recombination fraction analyses. There were 19 co-segregating marker pairs in F_2_ and thus only $1953 - 19 = 1934$ pairs of markers were used in the validation tests. Since the true recombination fractions of the marker pairs in the F_2_ generation were not known, we did not have the true recombination fractions to start with for calculating the theoretical recombination fractions for the F_3_ and F_4_ generations. We treated the observed recombination fractions for the F_2_ generation as the “true” values to calculate the theoretical recombination fractions of the F_3_ and F_4_ generations. To reduce the impact of the unknown initial recombination fractions of F_2_ on the theoretical values of the recombination fractions in F_3_ and F_4_, we took the average recombination fraction of all marker pairs with recombination fractions in the neighborhood of 0.05, 0.10, 0.15, 0.20, 0.25, 0.30, 0.35 and 0.40. The predicted recombination fractions of these marker pairs in the F_3_ and F_4_ generations were compared with the 95% confidence intervals (95% CIs) of the estimated recombination fractions. The estimated recombination fractions and the 95% CIs were calculated using the method described below.

Instead of directly estimating the recombination fractions between two markers using the expectation–maximization (EM) algorithm, we first estimated the correlation coefficient between the numerically coded genotypes (0, 1 and 2) of the two markers in the marker pair. Denote the estimated correlation coefficient between markers *i* and *j* by ${r_{ij}}$ with a standard error of

51 $${s_r} = \sqrt {\frac{{1 - r_{ij}^2}}{n - 2}}$$

where $n = 191$ is the sample size. The corresponding recombination fraction between the two markers is

52 $${\theta_{ij}} = \frac{1}{2}(1 - {r_{ij}})$$

with a standard error of ${\theta_{ij}}$ is

53 $${s_\theta } = \sqrt {\operatorname{var} ({\theta_{ij}})} = \sqrt {\frac{1}{4}\operatorname{var} ({r_{ij}})} = \frac{1}{2}{s_r}$$

The asymptotic 95% confidence interval is

54 $${\theta_{ij}} - 1.96{s_\theta } < {\theta_{ij}} < {\theta_{ij}} + 1.96{s_\theta }$$

**Fig. 7:**
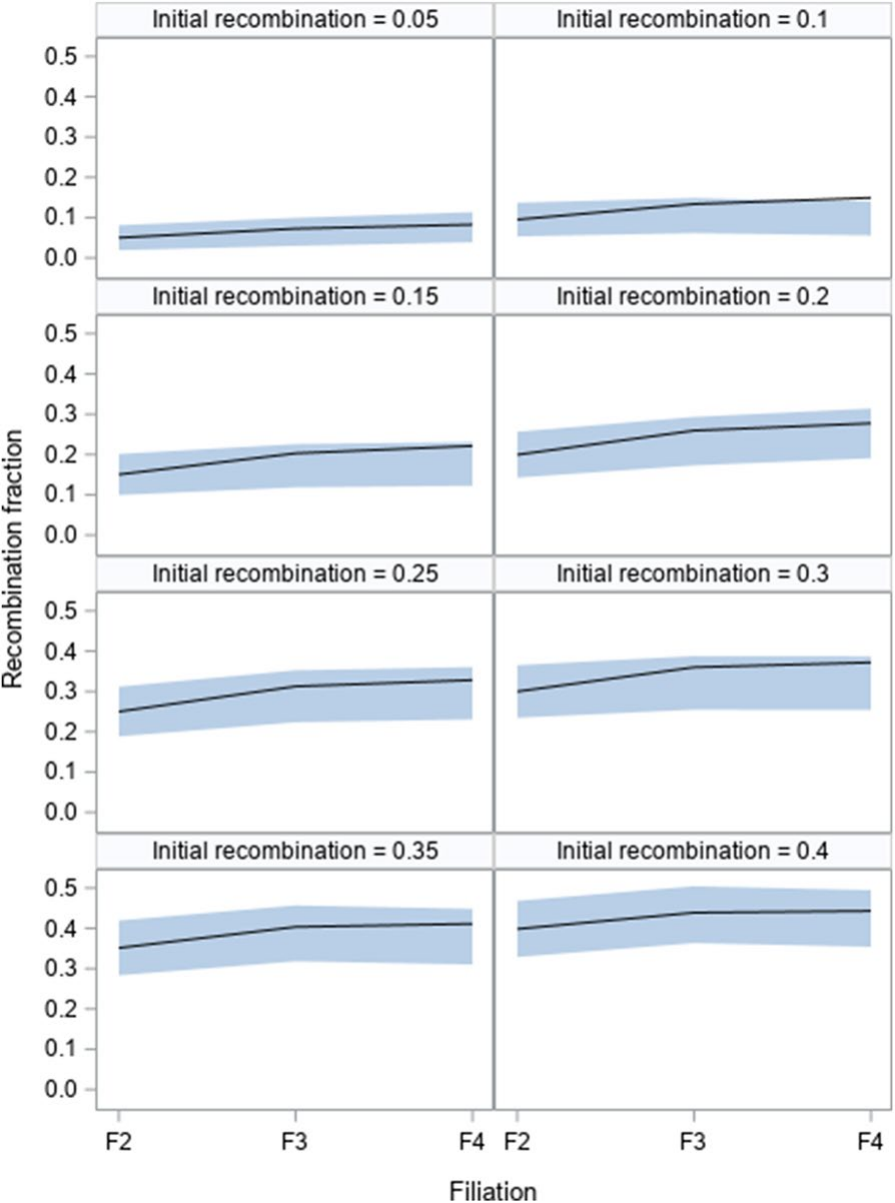
Predicted recombination fractions from the Markov model (solid lines) and the 95% confidence bands (light blue areas) from F_2_, F_3_ and F_4_ of a rice population

Figure 7 compares the theoretical recombination fractions (solid lines) calculated from the recurrent equations with the 95% confidence bands (light blue areas) of the estimated recombination fractions for F_2_, F_3_ and F_4_. The 95% confidence bands cover the theoretical recombinant fractions in all situations except F_4_ (the upper right panel) where the theoretical value barely touches the upper bound. The conclusion is that the theoretical recombination fractions calculated from the Markov model are valid.

## Discussion

The recurrent equations of genotype frequency array are Markov chains, which consist of two components: the probabilities of multiple states and the transition probabilities. Historically, the smaller the number of states, the easier the calculation. This was the very reason why Robbins [16] pooled the 16 fully phased genotypes of two loci into 10 unphased genotypes. Haldane and Waddington [6] further reduced the number of genotypes from 10 to 5. The reduction of the number of genotypes was very important in reducing the computational burdens in the pre-computer age. People can manually derive the transition probability matrix because of the lower dimension of the matrix. In the computer era, everything can be generated with computer code. The reduction of the number of genotypes is no longer important. We are dealing with a problem that the parameter (recombination fraction) is derived with recurrent equations, not estimated from observed data. Therefore, combining high dimensional fully phased genotypes into low dimensional unphased genotypes has lost its advantage. In fact, utilization of the fully phased genotypes with computer code can avoid human errors in manually writing the transition matrix.

An essential component of genetic mapping with RILs is to reconstruct the parental origins (the haplotypes) of DNA on the RIL chromosomes. In addition, QTL mapping using RILs as the genetic resources is a common practice in plants and small laboratory animals. With self-fertilization, as few as 8 generations are required [13]. How do we justify QTL mapping with PRERILs vs. RILs? Whether saving just a few of years using PRERILs for QTL mapping compared to using RILs is worth the effort considering the complexity of the mapping procedure. We argue that optimal utilization of the available genetic resources is always a factor to consider. If phenotypes and genotypes of PRERILs are available, why do we want to waste that information? QTL mapping with PRERILs may be important for laboratory animals because development of RILs requires about 20 generations of brother-sister mating. If we can perform QTL mapping with PRERILs half-way before RILs are fully developed, the time saved may be significant. The advanced intercross lines (AILs) increase the proportion of recombination between any two loci and thus provide precision to mapping closely linked QTL. The genetic basis of genome-wide association studies (GWAS) comes from the increased recombination fractions between loci.

Another justification for the study of recombination fraction in PRERILs is purely for scientific reason. We knew the recombination fraction both in the beginning (F_2_) and in the end (RILs) but did not know the trajectory how it reaches the equilibrium. This study for the first time fills the gap left for over 100 years.

There are many forms of repeated inbreeding. Jennings [7–9] investigated at least a dozen forms of them, including random mating, parent–offspring mating, assortative mating, self-fertilization, brother-sister mating, and selection with relation to one of the two loci. Robbins [14–16] reinvestigated majority of Jennings mating systems plus selection of dominants with respect to one of the two linked characters. Haldane and Waddington [6] investigate self-fertilization and parent–offspring mating with great details. Among all the mating systems, self-fertilization, and brother-sister mattings are the main forms of inbreeding to generate recombinant inbred lines.

In modern genetics, more advanced breeding systems have been developed for plants and laboratory animals, such as the Multi-parent Advanced Generation Inter-Cross (MAGIC) population in *Arabidopsis thaliana* [10] and the Collaboratory Crosses (CC) in mice [3]. The RILs of mice derived from an 8-way crosses of mice [2] were extension of the two -way cross of brother-sister mattings. Recurrent equations of genotype array and the recombination fraction between two loci in these complex inbreeding systems are difficult to derive. The number of genotype array can be huge, and the transition matrix may be in the order of thousand or ten thousand. Manual derivation is certainly not an option. If there is an interest, computer programs may be developed in the future to deal with the complex mating systems.

## Conclusions

We developed recurrent equations for calculating genotype frequencies for pre-recombinant inbred lines (PRERILs). These equations allow us to compute the recombination fractions between two loci before the lines reach the equilibrium state. An R function is provided for users to calculate the recombination fractions in PRERILs.

### Supplementary Information

Supplementary Material 1.

Supplementary Material 2.

Supplementary Material 3.

Supplementary Material 4.

Supplementary Material 5.

## Data Availability

Not applicable. The datasets used and/or analyzed during the current study are available from the corresponding author on reasonable request.

## Abbreviations

RIL: Recombinant inbred line
PRERIL: Pre-recombinant inbred line
QTL: Quantitative trait locus
AIL: Advanced intercross line

## Data availability

No datasets were generated or analysed during the current study.
