# Supplementary material for: Recombination fraction in pre-recombinant inbred lines (PRERIL) - revisiting a century old problem in genetics
Source: BMC Genomics. 2024 Sep 2;25:822. doi: 10.1186/s12864-024-10699-z (PMC11367787; doi:10.1186/s12864-024-10699-z)
Supplement: Supplementary file 2 — Supplementary Material 2. [file 12864_2024_10699_MOESM2_ESM.docx]

**Supplementary Note S1**

**Robbins’ recurrent equations for 10 unphased genotypes of self-fertilization**

Robbins (Robbins 1918) combined the same genotypes with different linkage phases together to form 10 distinguishable genotypes (out of 16 genotypes), as shown below,

$$\begin{matrix} c_{t}=ABAB \\ d_{t}=AbAb \\ e_{t}=aBaB \\ f_{t}=abab \\ g_{t}=ABab(AB/ab,ab/AB) \\ h_{t}=AbaB(Ab/aB,aB/Ab) \\ i_{t}=ABAb(AB/Ab,Ab/AB) \\ j_{t}=ABaB(AB/aB,aB/AB) \\ k_{t}=abAb(ab/Ab,Ab/ab) \\ l_{t}=abaB(ab/aB,aB/ab) \end{matrix}$$

The recombination fraction in the $\text{F}_{2}$ population is $\theta$, which is called the recombination fraction at generation $t=2$. Starting from $\text{F}_{1}$, the generation number is denoted by $t=1,2,\cdots,$ etc. Jennings (Jennings 1917), Robbins (Robbins 1918) and Haldane and Waddington (Haldane and Waddington 1931) denoted the generation number by $n$. However, *n* is often used as sample size. Therefore, we use generation index $t$ to denote the generation number to avoid any potential confusion. Robins (Robbins 1918) provided the following recurrent equations for the 10 unphased genotypes,

where

$$r=\frac{1}{\theta}(1-\theta)$$

and

$$R=4(1+r)^{2}=\frac{4}{\theta^{2}}$$

Substituting $r$ and $R$ in equation by equations and , respectively, we have

In the $\text{F}_{1}$ generation (generation 1), the genotype is $AB/ab$ and thus $g_{1}=1$ and all other genotypes have zero frequencies, i.e.,

$$c_{1}=d_{1}=e_{1}=f_{1}=h_{1}=i_{1}=j_{1}=k_{1}=l_{1}=0$$

Once we have established the recurrent equations and genotypic frequencies for generation $t$, we developed the probabilities of gametes as shown in **Table 1** below. The last column represents the probability of generating recombinant gametes from each genotype. There are four possible gametes (*AB, Ab, aB, ab*) that the 10 genotypes can produce. The numerical values of each of the four columns represent the probabilities that the genotypes can produce that kind of gametes. The sums of the two columns in the middle $(Ab+aB)$ represent the probabilities that the genotypes produce recombinant gametes. The last column (sum of the two columns in the middle) of the **Table 1** represents the weights of the genotypes to calculate the recombination fraction. Therefore, the recombination fraction at generation $t+1$ is

**Table 1** Gametic probabilities produced from the ten unphased genotypes at generation *t*.

| Entry | Genotype | Frequency | $AB$ | $Ab$ | $aB$ | $ab$ | $Ab+aB$ |
| --- | --- | --- | --- | --- | --- | --- | --- |
| 1 | $AABB$ | $c_{t}$ | 1 | 0 | 0 | 0 | 0 |
| 2 | $AAbb$ | $d_{t}$ | 0 | 1 | 0 | 0 | 1 |
| 3 | $aaBB$ | $e_{t}$ | 0 | 0 | 1 | 0 | 1 |
| 4 | $aabb$ | $f_{t}$ | 0 | 0 | 0 | 1 | 0 |
| 5 | $ABab$ | $g_{t}$ |  |  |  |  | $\theta$ |
| 6 | $AaBb$ | $h_{t}$ |  |  |  |  | $1-\theta$ |
| 7 | $AABb$ | $i_{t}$ | $\frac{1}{2}$ | $\frac{1}{2}$ | 0 | 0 | $\frac{1}{2}$ |
| 8 | $AaBB$ | $j_{t}$ | $\frac{1}{2}$ | 0 | $\frac{1}{2}$ | 0 | $\frac{1}{2}$ |
| 9 | $Aabb$ | $k_{t}$ | 0 | $\frac{1}{2}$ | 0 | $\frac{1}{2}$ | $\frac{1}{2}$ |
| 10 | $aaBb$ | $l_{t}$ | 0 | 0 | $\frac{1}{2}$ | $\frac{1}{2}$ | $\frac{1}{2}$ |

Equation says that the recombination fraction at generation $t+1$ is a linear function of the genotype frequencies of generation $t$. This is because the weight vector (the last column of **Table 1**) holds the frequencies of the gametes produced by generation *t*.

Robbins (Robbins 1918) eventually represented the genotypic frequencies as functions of the genotypic frequencies at generation 1 ($\text{F}_{1}$). Define

$$\begin{matrix} v=\frac{1}{2}\left[(1-\theta)^{2}+\theta^{2}\right] \\ w=\frac{1}{2}\left[(1-\theta)^{2}-\theta^{2}\right] \end{matrix}$$

The equation set for genotypes at generation *t* expressed as functions of genotypic frequencies at generation 1 are given in equations .

$$\begin{matrix} c_{t}=\frac{w(1-w^{t-1})}{4(1-w)}\left(g_{1}-h_{1}\right)+\frac{v^{t-1}-1}{4}\left(g_{1}+h_{1}\right)+\frac{2^{t-1}-1}{2^{t}}\left(g_{1}+h_{1}+i_{1}+j_{1}\right)+c_{1} \\ d_{t}=\frac{w(1-w^{t-1})}{4(1-w)}\left(h_{1}-g_{1}\right)+\frac{v^{t-1}-1}{4}\left(g_{1}+h_{1}\right)+\frac{2^{t-1}-1}{2^{t}}\left(g_{1}+h_{1}+i_{1}+k_{1}\right)+d_{1} \\ e_{t}=\frac{w(1-w^{t-1})}{4(1-w)}\left(h_{1}-g_{1}\right)+\frac{v^{t-1}-1}{4}\left(g_{1}+h_{1}\right)+\frac{2^{t-1}-1}{2^{t}}\left(g_{1}+h_{1}+j_{1}+l_{1}\right)+e_{1} \\ f_{t}=\frac{w(1-w^{t-1})}{4(1-w)}\left(g_{1}-h_{1}\right)+\frac{v^{t-1}-1}{4}\left(g_{1}+h_{1}\right)+\frac{2^{t-1}-1}{2^{t}}\left(g_{1}+h_{1}+k_{1}+l_{1}\right)+f_{1} \\ g_{t}=\frac{1}{2}w^{t-1}(g_{1}-h_{1})+\frac{1}{2}v^{t-1}(g_{1}+h_{1}) \\ h_{t}=\frac{1}{2}w^{t-1}(h_{1}-g_{1})+\frac{1}{2}v^{t-1}(g_{1}+h_{1}) \\ i_{t}=\frac{1}{2^{t}}\left(g_{1}+h_{1}+2i_{1}\right)-\frac{1}{2}v^{t-1}\left(g_{1}+h_{1}\right) \\ j_{t}=\frac{1}{2^{t}}\left(g_{1}+h_{1}+2j_{1}\right)-\frac{1}{2}v^{t-1}\left(g_{1}+h_{1}\right) \\ k_{t}=\frac{1}{2^{t}}\left(g_{1}+h_{1}+2k_{1}\right)-\frac{1}{2}v^{t-1}\left(g_{1}+h_{1}\right) \\ l_{t}=\frac{1}{2^{t}}\left(g_{1}+h_{1}+2l_{1}\right)-\frac{1}{2}v^{t-1}\left(g_{1}+h_{1}\right) \end{matrix}$$

At generation $t=1$, i.e., $g_{1}=1$ and all other genotypic frequencies are 0. Equation set becomes

$$\begin{matrix} c_{t}=\frac{w(1-w^{t-1})}{4(1-w)}+\frac{v^{t-1}-1}{4}+\frac{2^{t-1}-1}{2^{t}} \\ d_{t}=-\frac{w(1-w^{t-1})}{4(1-w)}+\frac{v^{t-1}-1}{4}+\frac{2^{t-1}-1}{2^{t}} \\ e_{t}=-\frac{w(1-w^{t-1})}{4(1-w)}+\frac{v^{t-1}-1}{4}+\frac{2^{t-1}-1}{2^{t}} \\ f_{t}=\frac{w(1-w^{t-1})}{4(1-w)}+\frac{v^{t-1}-1}{4}+\frac{2^{t-1}-1}{2^{t}} \\ g_{t}=\frac{1}{2}w^{t-1}+\frac{1}{2}v^{t-1} \\ h_{t}=-\frac{1}{2}w^{t-1}+\frac{1}{2}v^{t-1} \\ i_{t}=\frac{1}{2^{t}}-\frac{1}{2}v^{t-1} \\ j_{t}=\frac{1}{2^{t}}-\frac{1}{2}v^{t-1} \\ k_{t}=\frac{1}{2^{t}}-\frac{1}{2}v^{t-1} \\ l_{t}=\frac{1}{2^{t}}-\frac{1}{2}v^{t-1} \end{matrix}$$

Substituting all genotype frequencies in equation by the frequencies in equation leads to

$$\theta_{t+1}=1-\frac{w(1-w^{t-1})}{2(1-w)}+\frac{v^{t-1}-1}{2}+\frac{1}{2}\theta(w^{t-1}+v^{t-1})+\frac{1}{2}(1-\theta)(v^{t-1}-w^{t-1})-v^{t-1}$$

when $t=1$, $\theta_{t+1}=\theta_{2}$ and

$$\theta_{2}= 1-\frac{w(1-1)}{2(1-w)}+\frac{1-1}{2}+\frac{1}{2}\theta(1+1)+\frac{1}{2}(1-\theta)(1-1)-1=\theta$$

Therefore, the recombination fraction in the $\text{F}_{2}$ generation is $\theta$. When $t\to\infty$, $v^{\infty}=w^{\infty}=0$ and

$$\theta_{\infty}= 1-\frac{1}{2}-\frac{w}{2(1-w)}= \frac{1-\left[1-2\theta\right]}{2-\left[1-2\theta\right]}= \frac{2\theta}{1+2\theta}$$

This is the recombination fraction of the RIL population denoted by

$$\theta_{\infty}=\rho_{\text{self}}=\frac{2\theta}{1+2\theta}$$

**References**

Haldane JB, Waddington CH. 1931. Inbreeding and Linkage. *Genetics* **16**: 357-374.

Jennings HS. 1917. The Numerical Results of Diverse Systems of Breeding, with Respect to Two Pairs of Characters, Linked or Independent, with Special Relation to the Effects of Linkage. *Genetics* **2**: 97-154.

Robbins RB. 1918. Some Applications of Mathematics to Breeding Problems III. *Genetics* **3**: 375-389.
