# Supplementary material for: Recombination fraction in pre-recombinant inbred lines (PRERIL) - revisiting a century old problem in genetics
Source: BMC Genomics. 2024 Sep 2;25:822. doi: 10.1186/s12864-024-10699-z (PMC11367787; doi:10.1186/s12864-024-10699-z)
Supplement: Supplementary file 3 — Supplementary Material 3. [file 12864_2024_10699_MOESM3_ESM.docx]

**Supplementary Note S2**

**Haldane and Waddington’s recurrent equations for 5 composite genotypes**

**of self-fertilization**

We now review the recurrent equations developed by Haldane and Waddington (Haldane and Waddington 1931), who further reduced the number of genotypes from 10 to 5. They defined the recombination fraction by $\beta$ for the female gamete and $\delta$ for the male gamete. They defined the four gametes generated from the $\text{F}_{1}$ individual by

$$\begin{matrix} \text{Female}: (1-\beta)AB:(\beta)Ab:(\beta)aB:(1-\beta)ab \\ \text{Male}: (1-\delta)AB:(\delta)\text{ }Ab:(\delta)aB :(1-\delta)ab \end{matrix}$$

In this study, we assume that $\beta=\delta$=$\theta$ and replace $\beta$ and $\delta$ by $\theta$. The frequencies of the five genotypes defined by Haldane and Waddington (Haldane and Waddington 1931) are

$$\begin{matrix} C_{t}=AABB,aabb \\ D_{t}=AAbb,aaBB \\ E_{t}=AABb,AaBB,Aabb,aaBb \\ F_{t}=AB.ab \\ G_{t}=Ab.aB \end{matrix}$$

The recurrent equations (assuming $\theta=\beta=\delta$) are

The gametes produced by the genotypes have frequencies given in **Table 1**.

**Table 1** Gamete frequencies of the five genotypes at generation *t*.

| Genotype | Subtype | $AB$ | $Ab$ | $aB$ | $ab$ | $W=\text{Subtype}\times(Ab+aB)$ |
| --- | --- | --- | --- | --- | --- | --- |
| $C_{t}$ | 2 | 1/2 | 0 | 0 | 1/2 | 0 |
| $D_{t}$ | 2 | 0 | 1/2 | 1/2 | 0 | 2 |
| $E_{t}$ | 4 | 1/4 | 1/4 | 1/4 | 1/4 | 2 |
| $F_{t}$ | 1 |  |  |  |  | $\theta$ |
| $G_{t}$ | 1 |  |  |  |  | $1-\theta$ |

From Haldane and Waddington’s (Haldane and Waddington 1931) recurrent equations, we are able to derive equation for recombination fraction at generation $t+1$.

$$\begin{matrix} \theta_{t+1}= W_{1}C_{t}+W_{2}D_{t}+W_{3}E_{t}+W_{4}F_{t}+W_{5}G_{t} \\ = 2D_{t}+2E_{t}+\theta F_{t}+(1-\theta)G_{t} \end{matrix}$$

This is indeed much simpler than the equation from Robbins (Robbins 1918). Haldane and Waddington (Haldane and Waddington 1931) did not provide the result of the genotype frequencies at generation *t* expressed as functions of genotype frequencies at generation 1 (F_1_). However, they derived the stationary equilibrium value of the recombination fraction when the generation number is $t\to\infty$. We now present their derivation here for completeness of the study. They first defined $C_{t}-D_{t}=c_{t}$ and $F_{t}-G_{t}=d_{t}$. Then subtracted $D_{t+1}$ from $C_{t+1}$, leading to

This is simply rewritten as

Subtracting $G_{t+1}$ from $F_{t+1}$ leads to

which is rewritten as

Now choose $\lambda$ so that

$$c_{t+1}+\lambda d_{t+1}=c_{t}+\lambda d_{t}$$

for all values of $t$. Substituting $c_{t+1}$ and $d_{t+1}$ in equation by equations and , respectively, yields

Therefore,

Solving for $\lambda$ leads to

$$\lambda=\frac{1-2\theta}{2+4\theta}$$

Now, let us evaluate the genotype frequencies when $t=1$ (the initial generation) and when $t=\infty$(at the ultimate equilibrium). The initial population is the F_1_ hybrid with genotype $AB/ab$ or $AB.ab$ defined in equation . Therefore, in the initial population $F_{1}=2$ and $C_{1}=D_{1}=E_{1}=G_{1}=0$. This is due to an assumption of $2C_{t}+2D_{t}+4E_{t}+F_{t}+G_{t}=2$ (Haldane and Waddington 1931). In the final equilibrium state, all genotypes disappear except $C_{\infty}$ and $D_{\infty}$ remain. Therefore, $C_{\infty}+D_{\infty}=1$ and $E_{\infty}=F_{\infty}=G_{\infty}=0$. As a result, $d_{\infty}=F_{\infty}-G_{\infty}=0-0=0$, $c_{1}=C_{1}-D_{1}=0-0=0$, and $d_{1}=F_{1}-G_{1}=2-0=2$. Given the values in the special case, we have

$$c_{\infty}=c_{\infty}+\lambda d_{\infty}=c_{1}+\lambda d_{1}=2\lambda=2\times\frac{1-2\theta}{2+4\theta}=\frac{1-2\theta}{1+2\theta}$$

At the equilibrium state, we have the following two equations,

$$\begin{matrix} C_{\infty}+D_{\infty}=1 \\ C_{\infty}-D_{\infty}=c_{\infty} \end{matrix}$$

Subtracting the second equation of from the first equation of leads to

$$2D_{\infty}=1-c_{\infty}$$

Therefore,

$$D_{\infty}=\frac{1}{2}(1-c_{\infty})=\frac{1}{2}\left(1-\frac{1-2\theta}{1+2\theta}\right)=\frac{1}{2}\left(\frac{1+2\theta-1+2\theta}{1+2\theta}\right)=\frac{2\theta}{1+2\theta}$$

Since $D_{\infty}$ is the proportion of the recombinant gametes at the equilibrium state, we have the final solution,

$$\theta_{\infty}=\rho_{\text{self}}=\frac{2\theta}{1+2\theta}$$

**References**

Haldane JB, Waddington CH. 1931. Inbreeding and Linkage. *Genetics* **16**: 357-374.

Robbins RB. 1918. Some Applications of Mathematics to Breeding Problems III. *Genetics* **3**: 375-389.
