# Supplementary material for: Recombination fraction in pre-recombinant inbred lines (PRERIL) - revisiting a century old problem in genetics
Source: BMC Genomics. 2024 Sep 2;25:822. doi: 10.1186/s12864-024-10699-z (PMC11367787; doi:10.1186/s12864-024-10699-z)
Supplement: Supplementary file 4 — Supplementary Material 4. [file 12864_2024_10699_MOESM4_ESM.docx]

**Supplementary Note S3**

**Recurrent equations of gametes of random mating**

**1 Robbins derivation**

Robbins (Robbins 1918) defined the four gametic frequencies at generation *t* by $p_{t}(AB)$, $q_{t}(Ab)$, $s_{t}(aB)$ $t_{t}(ab)$. Define $r$ as the linkage parameter, which is related to the recombination fraction by

$$r=(1-\theta)/\theta$$

or

$$\theta=1/(1+r)$$

where $\theta$ is the recombination fraction in modern genetics. Robbins adopted Jennings’s (Jennings 1917) recurrent equations for the four gametic frequencies,

$$\begin{matrix} p_{t+1}= (r+1)p_{t}(p_{t}+q_{t}+s_{t})+rp_{t}t_{t}+q_{t}s_{t} \\ q_{t+1}= (r+1)q_{t}(p_{t}+q_{t}+t_{t})+rq_{t}s_{t}+p_{t}t_{t} \\ s_{t+1}= (r+1)s_{t}(p_{t}+s_{t}+t_{t})+rq_{t}s_{t}+p_{t}t_{t} \\ t_{t+1}= (r+1)t_{t}(q_{t}+s_{t}+t_{t})+rp_{t}t_{t}+q_{t}s_{t} \end{matrix}$$

These recurrent equations are non-linear and thus are not Markov chains. However, because there are only four gametes, the recurrent equations are manually derivable. Let us define

$$\Delta_{t}=q_{t}s_{t}-p_{t}t_{n}$$

Robbins (Robbins 1918) rewrote equation by

$$\begin{matrix} p_{t}= p_{t-1}+\Delta_{t-1}/(1+r) \\ q_{t}= q_{t-1}-\Delta_{t-1}/(1+r) \\ s_{t}= s_{t-1}-\Delta_{t-1}/(1+r) \\ t_{t}= t_{t-1}+\Delta_{t-1}/(1+r) \end{matrix}$$

Eventually, the gametic frequencies at generation *t* are expressed as functions of the gametic frequencies at generation 1.

Since

$$\frac{r}{1+r}=\frac{(1-\theta)/\theta}{1+(1-\theta)/\theta}=\frac{1-\theta}{\theta+(1-\theta)}=1-\theta$$

Equation is rewritten as

$$\begin{matrix} p_{t}= p_{1}+\Delta_{1}\left[1-\left(1-\theta\right)^{t-1}\right] \\ q_{t}= q_{1}-\Delta_{1}\left[1-\left(1-\theta\right)^{t-1}\right] \\ s_{t}= s_{1}-\Delta_{1}\left[1-\left(1-\theta\right)^{t-1}\right] \\ t_{t}= t_{1}+\Delta_{1}\left[1-\left(1-\theta\right)^{t-1}\right] \end{matrix}$$

Note that the generation index *t* defined in gametes differs from the generation index in genotypes by 1, i.e., gametic generation *t* means genotype generation $t+1$. At generation 1 ($\text{F}_{1}$), the genotype is $AB/ab$ and the frequencies of the four gametes from the $\text{F}_{1}$ hybrid are

Therefore,

$$\Delta_{1}=q_{1}s_{1}-p_{1}t_{1}=\frac{1}{4}\left[\theta^{2}-(1-\theta)^{2}\right]=\frac{1}{4}(2\theta-1)$$

The frequencies of the recombinant gametes at generation *t* is

$$\begin{matrix} \theta_{t}= q_{t}+s_{t} \\ = q_{1}+s_{1}-2\Delta_{1}\left[1-(1-\theta)^{t-1}\right] \\ = \theta-2\times\frac{1}{4}(2\theta-1)\left[1-(1-\theta)^{t-1}\right] \\ = \theta-\frac{1}{2}(2\theta-1)\left[1-(1-\theta)^{t-1}\right] \\ = \frac{1}{2}\left[1+(2\theta-1)(1-\theta)^{t-1}\right] \end{matrix}$$

Deleting the intermediate steps leads to

$$\theta_{t}= \frac{1}{2}\left[1+(2\theta-1)(1-\theta)^{t-1}\right]$$

**2 Darvasi and Soller’s approach**

Surprisingly, Darvasi and Soller (Darvasi and Soller 1995) used an extremely simple method to derive the same formula. They set the recombination fraction at generation $t-1$ to $\theta_{t-1}$. The recombination fraction of the next generation is $\theta_{t}$, which is contributed by $\theta_{t-1}$ plus the gain from the recombinants produced by $AB/ab$ and $ab/AB$, and minus the loss of recombinants produced by crossovers by $Ab/aB$ and $aB/Ab$. From **Table 1** of the main text, we found that the probability of $AB/ab$ and $ab/AB$ is

$$\frac{1}{4}(1-\theta_{t-1})^{2}+\frac{1}{4}(1-\theta_{t-1})^{2}=\frac{1}{2}(1-\theta_{t-1})^{2}$$

If recombination happens to $AB/ab$ and $aB/Ab$, the gain of the recombinants is

$$\text{gain}_{\theta}=\frac{1}{2}\theta(1-\theta_{t-1})^{2}$$

If recombination happens to $Ab/aB$ and $aB/Ab$, they generate the parental types and thus the loss of recombinants is

$$\text{loss}_{\theta}=\frac{1}{2}\theta\theta_{t-1}^{2}$$

Therefore,

$$\theta_{t}=\theta_{t-1}+\text{gain}_{\theta}-\text{loss}_{\theta}=\theta_{t-1}+\frac{1}{2}\theta(1-\theta_{t-1})^{2}-\frac{1}{2}\theta\theta_{t-1}^{2}=\frac{1}{2}\theta+\theta_{t-1}(1-\theta)$$

Substituting $\theta_{t-1}$ by

$$\theta_{t-1}=\frac{1}{2}\theta+\theta_{t-2}(1-\theta)$$

leads to

$$\theta_{t}=\frac{1}{2}\theta+\theta_{t-1}(1-\theta)=\frac{1}{2}\theta+\left[\frac{1}{2}\theta+\theta_{t-2}(1-\theta)\right](1-\theta)$$

This process continues until $\theta_{t}$ is expressed as a function of the initial recombination fraction,

$$\theta_{t}=\frac{1}{2}\left[1-(1-\theta)^{t-1}(1-2\theta)\right]$$

which is identical to equation derived from the Robbins’ (Robbins 1918) recurrent equations. For $t=1$, $\theta_{1}=\theta$ while $\theta_{\infty}=0.5$ for $t\to\infty$.

**References**

Darvasi A, Soller M. 1995. Advanced intercross lines, an experimental population for fine genetic mapping. *Genetics* **141**: 1199-1207.

Jennings HS. 1917. The Numerical Results of Diverse Systems of Breeding, with Respect to Two Pairs of Characters, Linked or Independent, with Special Relation to the Effects of Linkage. *Genetics* **2**: 97-154.

Robbins RB. 1918. Some Applications of Mathematics to Breeding Problems III. *Genetics* **3**: 375-389.
