# Supplementary material for: Recombination fraction in pre-recombinant inbred lines (PRERIL) - revisiting a century old problem in genetics
Source: BMC Genomics. 2024 Sep 2;25:822. doi: 10.1186/s12864-024-10699-z (PMC11367787; doi:10.1186/s12864-024-10699-z)
Supplement: Supplementary file 5 — Supplementary Material 5. [file 12864_2024_10699_MOESM5_ESM.docx]

**Supplementary Table S1** Transition probability table (the *P* matrix) for the 16 genotypes of self-fertilization.

|  | G1 | G2 | G3 | G4 | G5 | G6 | G7 | G8 | G9 | G10 | G11 | G12 | G13 | G14 | G15 | G16 |
| --- | --- | --- | --- | --- | --- | --- | --- | --- | --- | --- | --- | --- | --- | --- | --- | --- |
| G1 | 1 | $\frac{1}{4}$ | $\frac{1}{4}$ |  | $\frac{1}{4}$ | 0 |  | 0 | $\frac{1}{4}$ |  | 0 | 0 |  | 0 | 0 | 0 |
| G2 | 0 | $\frac{1}{4}$ | 0 |  | $\frac{1}{4}$ | 0 |  | 0 | 0 |  | 0 | 0 |  | 0 | 0 | 0 |
| G3 | 0 | 0 | $\frac{1}{4}$ |  | 0 | 0 |  | 0 | $\frac{1}{4}$ |  | 0 | 0 |  | 0 | 0 | 0 |
| G4 | 0 | 0 | 0 |  | 0 | 0 |  | 0 | 0 |  | 0 | 0 |  | 0 | 0 | 0 |
| G5 | 0 | $\frac{1}{4}$ | 0 |  | $\frac{1}{4}$ | 0 |  | 0 | 0 |  | 0 | 0 |  | 0 | 0 | 0 |
| G6 | 0 | $\frac{1}{4}$ | 0 |  | $\frac{1}{4}$ | 1 |  | $\frac{1}{4}$ | 0 |  | 0 | 0 |  | $\frac{1}{4}$ | 0 | 0 |
| G7 | 0 | 0 | 0 |  | 0 | 0 |  | 0 | 0 |  | 0 | 0 |  | 0 | 0 | 0 |
| G8 | 0 | 0 | 0 |  | 0 | 0 |  | $\frac{1}{4}$ | 0 |  | 0 | 0 |  | $\frac{1}{4}$ | 0 | 0 |
| G9 | 0 | 0 | $\frac{1}{4}$ |  | 0 | 0 |  |  | $\frac{1}{4}$ |  | 0 | 0 |  |  | 0 | 0 |
| G10 | 0 | 0 | 0 |  | 0 | 0 |  | 0 | 0 |  | 0 | 0 |  | 0 | 0 | 0 |
| G11 | 0 | 0 | $\frac{1}{4}$ |  | 0 | 0 |  | 0 | $\frac{1}{4}$ |  | 1 | $\frac{1}{4}$ |  | 0 | $\frac{1}{4}$ | 0 |
| G12 | 0 | 0 | 0 |  | 0 | 0 |  | 0 | 0 |  | 0 | $\frac{1}{4}$ |  | 0 | $\frac{1}{4}$ | 0 |
| G13 | 0 | 0 | 0 |  | 0 | 0 |  | 0 | 0 |  | 0 | 0 |  | 0 | 0 | 0 |
| G14 | 0 | 0 | 0 |  | 0 | 0 |  | $\frac{1}{4}$ | 0 |  | 0 | 0 |  | $\frac{1}{4}$ | 0 | 0 |
| G15 | 0 | 0 | 0 |  | 0 | 0 |  | 0 | 0 |  | 0 | $\frac{1}{4}$ |  | 0 | $\frac{1}{4}$ | 0 |
| G16 | 0 | 0 | 0 |  | 0 | 0 |  | $\frac{1}{4}$ | 0 |  | 0 | $\frac{1}{4}$ |  | $\frac{1}{4}$ | $\frac{1}{4}$ | 1 |
